# Flavonoids as Potential Modulators of Pancreatic Lipase Catalytic Activity

**DOI:** 10.3390/pharmaceutics17020163

**Published:** 2025-01-25

**Authors:** Sílvia Rocha, Carina Proença, Alberto N. Araújo, Marisa Freitas, Ismael Rufino, Natália Aniceto, Artur M. S. Silva, Félix Carvalho, Rita C. Guedes, Eduarda Fernandes

**Affiliations:** 1 Laboratório Associado para a Química Verde (LAQV), Rede de Química e Tecnologia (REQUIMTE), Laboratory of Applied Chemistry, Department of Chemical Sciences, Faculty of Pharmacy, University of Porto, Rua de Jorge Viterbo Ferreira nº 228, 4050-313 Porto, Portugal; up200702315@ff.up.pt (S.R.); cproenca@ff.up.pt (C.P.); anaraujo@ff.up.pt (A.N.A.); marisafreitas@ff.up.pt (M.F.); 2 Research Institute for Medicines (iMed.ULisboa), Faculty of Pharmacy, University of Lisboa, Av. Prof. Gama Pinto, 1649-003 Lisboa, Portugal; ismaelcarvalho@edu.ulisboa.pt (I.R.); nataliaaniceto@ff.ulisboa.pt (N.A.); 3 Laboratório Associado para a Química Verde (LAQV), Rede de Química e Tecnologia (REQUIMTE), Department of Chemistry, University of Aveiro, 3800-193 Aveiro, Portugal; artur.silva@ua.pt; 4 UCIBIO—Applied Molecular Biosciences Unit, Laboratory of Toxicology, Department of Biological Sciences, Faculty of Pharmacy, University of Porto, 4050-313 Porto, Portugal; felixdc@ff.up.pt; 5 Associate Laboratory i4HB—Institute for Health and Bioeconomy, Faculty of Pharmacy, University of Porto, 4050-313 Porto, Portugal

**Keywords:** obesity, pancreatic lipase inhibition, in vitro, in silico, flavonoids, structure–activity relationship

## Abstract

**Background/Objectives**: Obesity has reached pandemic proportions, with predictions suggesting that, by 2030, over 1.5 billion people will be affected. Pancreatic lipase (PL), the enzyme primarily responsible for the absorption of dietary lipids, presents a potential target for obesity management. However, while porcine pancreatic lipase (PPL) is commonly used as the enzyme source for screening potential inhibitors, its effect on human pancreatic lipase (HPL) is rarely reported. This work aimed to screen the inhibitory effects of a library of flavonoids with different functional groups on the activity of PL from the human pancreas (triacylglycerol acyl hydrolase, EC 3.1.1.3) and compare it to the effects of the porcine pancreas (type II, EC 3.1.1.3), establishing, whenever possible, a structure–activity relationship. **Methods**: The inhibitory effects of a library of 48 flavonoids with different hydroxy, glycosyl, rutinosyl, galloyl, and extended alkyl groups were evaluated against PPL and HPL. The kinetic parameters and inhibitory mechanisms of the most active flavonoids were determined, and in silico docking studies of the more potent flavonoids were also performed, using the active site of HPL. **Results/Conclusions**: Variations in enzyme catalytic activity were observed depending on the source of the enzyme. The inhibitory effect was particularly influenced by the presence of extended alkyl groups at the C-3 of the C-ring and the C2=C3 double bond of the C-ring and the presence of a pyrogallol group at the C-2′, C-3′ and C-4′ of the B-ring. Docking results showed a strong correlation between docking scores and observed inhibitory activities, highlighting the critical role of specific substituents on the flavonoid backbone in enhancing detailed interaction dynamics with key amino acids. Compounds 28, 29, and 30, with alkyl groups, showed the highest docking scores, interacting with residues HIS151, PHE215, ARG256, and HIS263. Further analysis also revealed that specific substituents improved pocket occupancy and formed additional interactions with residues TYR114, PRO180, ILE209, and PHE215, which are crucial for inhibition. These binding characteristics closely mimic those observed with orlistat, reinforcing their mechanistic similarities in inhibiting HPL and validating their inhibitory activities.

## 1. Introduction

The World Health Organization (WHO) defines obesity as an excessive accumulation of body fat, indicated by a Body Mass Index (BMI) ≥ 30 kg/m^2^ (person’s weight/height^2^) [1]. Further considering etiologic aspects, the World Obesity Federation (WOF) classifies obesity as a chronic, relapsing, and progressive disease, resulting from a long-term energy imbalance between calories consumed and expended. This imbalance is influenced by poor nutrition, unhealthy lifestyle choices, and various biological, psychosocial, and behavioral factors, including genetic, socioeconomic, and cultural influences [2,3,4].

Over the past fifty years, the morbidity associated with obesity and the corresponding healthcare costs have escalated to pandemic proportions [3,5]. In 2010, nearly 15% of the global population was obese and, according to the latest WOF predictions, by 2035, this figure is expected to rise to 25%, representing over 1.9 billion people. Alarmingly, by 2035, nearly 400 million children and adolescents aged 5 to 19 years are predicted to be affected by obesity [6,7].

Obesity is linked directly or indirectly to an increased risk of metabolic and cardiovascular diseases, depression, and certain types of cancer. This connection results in high mortality rates, reduced life expectancy, and a diminished quality of life, impacting educational attainment and social interactions [4,5,8]. Different approaches are available to prevent and treat obesity. Physical activity is highly recommended and effective [9,10], and daily diet planning is crucial [11]. Treatment strategies depend on the individual’s BMI, and their application takes into account its cost, effectiveness, and, most importantly, its safety [12]. One strategy involves developing inhibitors of nutrient digestion and absorption to reduce energy intake. Since most unwanted calories come from lipids, inhibiting fat absorption is a promising approach [13]. In the small intestine, bile emulsifies large fat globules, readily breaking them into smaller pieces that pancreatic lipase (PL) can hydrolyze into fatty acids before absorption. PL is responsible for hydrolyzing 50 to 70% of total dietary fats, making its inhibition a key target for reducing fat intake [14].

Human pancreatic lipase (HPL) shares 85% sequence identity in its coding region with the protein sequence of porcine pancreatic lipase (PPL) [15,16]. PPL’s high degree of similarity, coupled with its lower acquisition cost, supports its prevalent use in scientific research. However, the literature contains discrepancies in inhibitor responses when using both enzymes [17,18]. Hence, understanding the correlation between the inhibitor potency exhibited in PPL and HPL is pivotal for identifying more effective inhibitors, using HPL as the enzyme source to improve the translational applicability of the research findings.

Currently, orlistat (commercially known as Xenical^®^, whose trademark is owned by F. Hoffmann-La Roche AG, city, Country) is the only approved drug for obesity treatment that acts as a PL inhibitor. It is also the only anti-obesity drug prescribed for adolescents and the only anti-obesity drug that does not involve appetite suppression or direct effects on the central nervous system, unlike other medications such as Ozempic [19,20]. However, orlistat is associated with several gastrointestinal side effects and the potential malabsorption of fat-soluble vitamins and other essential nutrients, potentially causing nutritional deficiencies if not monitored and supplemented properly. While these side effects are linked to the mechanism of action of orlistat as a lipase inhibitor, it remains unclear whether they are solely a result of PL inhibition, or if other factors contribute to these effects. This uncertainty underscores the need to explore alternative PL inhibitors with improved safety profiles to maintain efficacy while mitigating adverse side effects [21].

Flavonoids, dietary secondary metabolites found abundantly in vegetables and fruits, are believed to possess health-promoting properties in both in vitro and in vivo systems [22]. This is supported by their ability to induce human protective enzyme systems, with several epidemiological studies highlighting their protective effects, such as anti-inflammatory, antioxidant, antidiabetic, and anti-obesity activities [23,24]. However, the potential health benefits of flavonoids depend on their intake and bioavailability, which can vary significantly due to factors such as rapid metabolism and fast elimination, thereby limiting their therapeutic potential [25]. On the other hand, some potentially toxic effects have also been reported. While these findings are relevant, there are still many contradictions across different epidemiological investigations. Flavonoid extracts are frequently tested in vitro and in vivo using animal models, while abundant dietary flavonoids, such as those found in soybeans, are more commonly investigated in clinical trials. One of the main challenges is the difficulty of translating the concentrations used in in vitro studies to human-relevant doses. Moreover, due to the complexity of food composition and the metabolic transformations that occur in the digestive system, it is often unclear whether the observed toxicity is directly linked to the parent flavonoid structures or to other metabolites [25,26]. As a result, the current data on flavonoids’ toxicity remains inconclusive. Further research is essential to clarify these discrepancies, overcome the existing limitations, and fully realize the therapeutic potential of flavonoids, particularly in terms of their clinical application.

**Figure 1:**
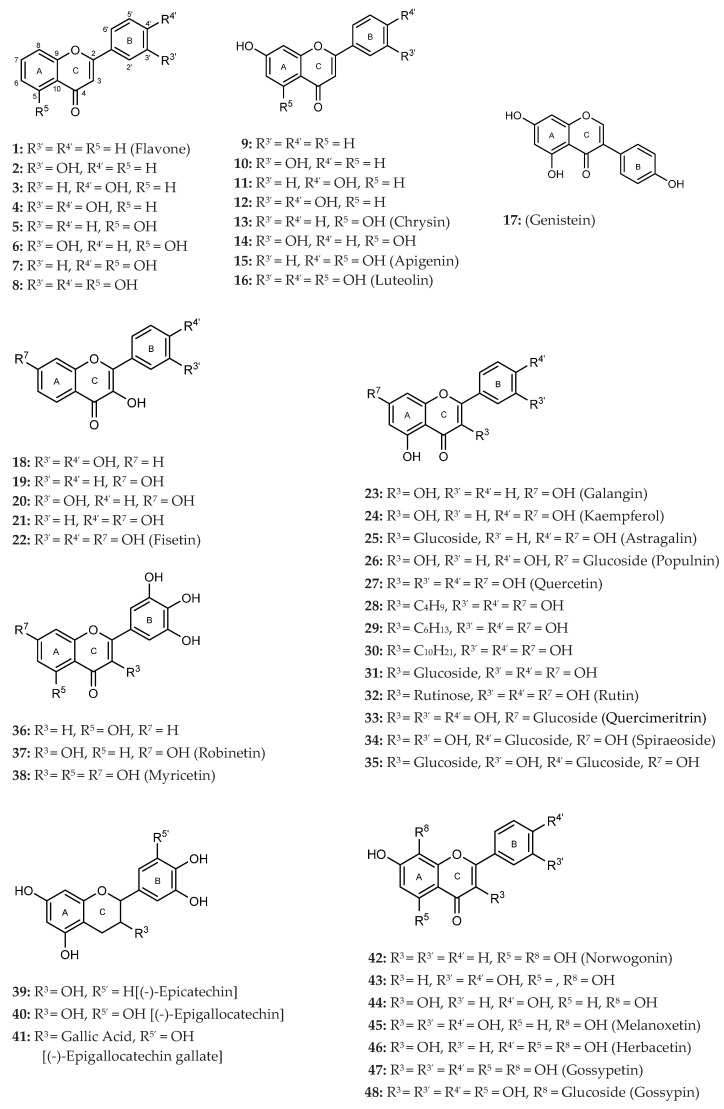
Chemical structure of the studied flavonoids.

To make meaningful progress in scientific research, it is crucial to first identify and deeply understand which flavonoids are worthy of investigation. This foundational step is essential, as it allows us to prioritize those flavonoids that exhibit the most promising biological activities, therapeutic potential, or relevance to the specific health issues being addressed, particularly anti-obesity effects. Despite several studies that have explored flavonoids as potential PL inhibitors, a systematic structure–activity relationship remains underexplored, due to varying methodologies and experimental conditions in previous studies [27]. Therefore, 48 flavonoids (Figure 1) were evaluated in this study, regarding their potential as PL inhibitors, to establish a structure–activity relationship. An in vitro microanalysis screening system using PPL and HPL is described, and the kinetic inhibition mechanisms of the most active compounds were assessed. Additionally, to clarify the structure–activity relationship in this new series of potential inhibitors, in silico docking studies of the more potent flavonoids were also performed using the active site of HPL.

## 2. Materials and Methods

### 2.1. Chemicals

Sodium chloride (NaCl) and dimethyl sulfoxide (DMSO) were obtained from Fisher Scientific Inc. (Loughborough, UK), and tris(hydroxymethyl)aminomethane (Tris base) was obtained from NZYTech, Lda. (Lisbon, Portugal). Calcium chloride (CaCl_2_), 4-methylumbelliferyl oleate (4-MUO), PL (type II) from porcine pancreas, PL from human pancreas (triacylglycerol acyl hydrolase, EC3.1.1.3), orlistat, and flavonoids **15**, **27**, **39,** and **41** were obtained from Sigma-Aldrich Co. LLC (St. Louis, MO, USA). Flavonoids **16** and **17** were obtained from ThermoFisher Scientific (Waltham, MA, USA) and Santa Cruz Biotechnology (Dallas, TX, USA), respectively, and flavonoids **22**, **31,** and **44** were obtained from Biosynth Ltd. (Berkshire, UK). Flavonoids **19**, **24–26**, **32–35**, **37**, **40**, **42,** and **46** were provided by Extrasynthese (Lyon Nord, France) and the following flavonoids were obtained from Indofine Chemical Company, Inc. (Hillsborough, NJ, USA): **1–13**, **18**, **20**, **21**, **23**, **38**, **43**, **47,** and **48**. The remaining flavonoids **14**, **28–30**, **36,** and **45** were synthesized according to previously published methods [28,29,30].

### 2.2. In Vitro Assay Using Porcine Pancreatic Lipase

The assay using the PPL enzyme was based on the method described in a previous publication [31], with some modifications. In each assay, PPL and 4-MUO served as the reaction enzyme and fluorogenic substrate, respectively, and the PPL-mediated hydrolysis of the 4-MUO into 4-methylumbeliferone (4-MU) was monitored. The initial rate of 4-MU generation, measured fluorometrically, was proportional to the concentration of PPL present. The intrinsic fluorescence of each compound was previously tested under the assay conditions.

In brief, in a black 96-well plate, 200 μL of the enzyme (final concentration 0.04 mg/mL), dissolved in Tris buffer (13 mM Tris, 150 mM NaCl, and 1.3 mM CaCl_2_, pH 7.4), was pre-incubated with 10 μL of the flavonoids under study (**1** to **48**, 0–100 μM), and dissolved in DMSO [the final concentration of DMSO (4% *v*/*v*) did not interfere with the assay]. After a pre-incubation time of 15 min, at 37 °C, the reaction was started by the addition of 40 μL of 4-MUO (final concentration 0.05 mM), dissolved in 13 mM Tris buffer (pH 7.4) and continuously monitored for 30 min in a monochromator microplate reader (Cytation 5, BIO-TEK, Winooski, VT, USA). The fluorescence was measured at the excitation wavelength of 405 ± 10 nm and emission wavelength of 450 ± 25 nm. Wavelength values and bandwidth were optimized to improve signal response and minimize possible interferences.

Orlistat (0–30 nM) was used as the positive control.

The slopes describing the relative fluorescence unit (RFU) values increase within 10 to 30 min intervals (slope) served for the calculation of catalytic rates, and the results are presented as inhibition percentage, representing at least three independent experiments.

### 2.3. In Vitro Assay Using Human Pancreatic Lipase

The HPL inhibition assay was based on the method described in a previous publication [32], with slight modifications. Similarly to porcine enzymatic assay, HPL and 4-MUO served as the reaction enzyme and fluorogenic substrate, respectively, and the HPL-mediated hydrolysis of the 4-MUO into 4-MU was monitored. The initial rate of 4-MU generation, measured fluorometrically, was proportional to the concentration of HPL present.

In a black 96-well plate, 50 μL of the enzyme (final concentration 2 U/mL), dissolved in 1.3 mM Tris buffer (pH 7.4), was pre-incubated with 25 μL of the flavonoids under study (**1** to **48**, 0–100 μM), and dissolved in DMSO [the final concentration of DMSO [20% (*v*/*v*) did not interfere with the assay]. After a pre-incubation time of 15 min, at 37 °C, the reaction was started by the addition of 40 μL of 4-MUO (final concentration 0.05 mM), dissolved in Tris buffer (13 mM Tris, 150 mM NaCl, and 1.3 mM CaCl_2_, pH 7.4), and continuously monitored for 30 min in a monochromator microplate reader. The fluorescence was measured at the same wavelengths as for PPL (excitation wavelength of 405 ± 10 nm and emission wavelength of 450 ± 25 nm), and the calculation of catalytic rates was performed as described in the PPL inhibition assay.

Orlistat (0–2 nM) was used as the positive control.

### 2.4. Inhibition Kinetic of Pancreatic Lipase

The kinetic parameters and the prediction of the PPL and HPL catalytic activity inhibition were performed using Microsoft Office Excel 365^TM^ spreadsheets, along with Solver supplement Add-in, according to Freitas et al. [33]. In brief, to study PPL and HPL catalytic activity and calculate the Michaelis–Menten constant, *K_m_* (expressed in mM)*,* the competitive and uncompetitive constants, *K_ic_* and *K_iu_*, respectively (both expressed in μM^−1^), the kinetics conversion of 4-MUO by PPL, and HPL in each microplate well, were fitted by nonlinear least squares regression using the general model for enzymatic reactions via the well-known Michaelis–Menten Equation (1), as well as its modified forms (2) to (5) in the presence of the putative inhibitor, regarding the different types of inhibition:

(1) $$v_{\mathrm{inic}}=\frac{V_{\max}\;\times \;|S|}{K_{m}+|S|}\;\;(\mathrm{without}\;\mathrm{inhibition})$$

(2) $$v_{\mathrm{inic}}=\frac{V_{\max\;}\times \;|S|}{K_{m\;\times \;}\alpha_{\mathrm{ic}}+|S|}\;\;(\mathrm{competitive}\;\mathrm{inhibition})$$

(3) $$v_{\mathrm{inic}}=\frac{V_{\max}\;{/\;\alpha }_{\mathrm{iu}}\times \;|S|}{K_{m\;}/\;\alpha_{\mathrm{iu}\;}+\left|S\right|}\;\;(\mathrm{noncompetitive}\;\mathrm{inhibition})$$

(4) $$v_{inic}=\frac{V_{max}\;{/\;\alpha }_{iu}\times |S|}{K_{m}\times \alpha_{ic}{\;/\;\alpha }_{iu}+\left|S\right|}\;\;(\mathrm{uncompetitive}\;\mathrm{inhibition})$$

(5) $$v_{inic}=\frac{V_{max}\;/\;\alpha_{iu}\times |S|}{K_{m}\times \alpha_{in}+\left|S\right|}\;\;(\mathrm{mixed}\;\mathrm{inhibition})$$

$$\mathrm{with}:\;\alpha_{\mathrm{ic}}=\left(1+\frac{\left|I\right|}{K_{\mathrm{ic}}}\right),\;\alpha_{\mathrm{iu}}=\left(1+\frac{\left|I\right|}{K_{\mathrm{iu}}}\right)\;\mathrm{and}\;\alpha_{\mathrm{in}}=\alpha_{\mathrm{ic}}/\alpha_{\mathrm{iu}}\approx 1$$

where ν_inic_ is the initial velocity of formation of 4-MU in μmol/min, V_max_ is the maximum achievable velocity when all catalytic sites for the enzyme (0.04 mg/mL for PPL and 2 U/mL for HPL) are saturated by the substrate when the inhibitor is not used, |S| is the concentration of 4-MUO in μM, and |I| is the concentration of the inhibitor in μM. Each analysis was performed with and without the inhibitor. Additionally, double reciprocal plots for each concentration of both inhibitor and substrate were also built to visualize how the fitted models stay apart from the experimental data points.

To study enzyme kinetics, nonlinear regression was applied to the non-transformed data with and without orlistat and the most active flavonoids (Table 1). For each concentration of inhibitor, three concentrations of the substrate 4-MUO were tested. The obtained results represent at least three independent experiments.

The Solver tool was used to iteratively minimize the sum of squared residuals between the experimental values of V_max_ and the corresponding estimated values for each tested condition, to obtain the best guesses for the equation parameters for each condition. The simplest model, without inhibition, was used to obtain initial guesses of V_max_ and K_m_. The parameter values for the more complex models, including competitive inhibition, noncompetitive inhibition, uncompetitive inhibition, and mixed inhibition, were then determined in sequence.

The correct model was selected by performing a simplified comparison of the sum of squared residuals, obtained by applying the Solver tool for the different models. To determine the error of the kinetic constant values, the jackknife procedure was applied through the standard deviation determination of all estimates guessed by Solver when each experimental data point was sequentially removed from the initial set.

### 2.5. Statistical Analysis

The in vitro inhibitory effects of the compounds against PPL and HPL were expressed as IC_50_ (half-maximal inhibitory concentration) or % of inhibition at a defined concentration, both as mean ± standard error of the mean (SEM). Statistical differences were considered significant at values lower than 0.05. All the statistical analyses were performed using GraphPad Prism^TM^ (version 9.0, GraphPad Software, Boston, MA, USA).

### 2.6. Computational Studies

#### 2.6.1. Protein Preparation and Binding Pocket Analysis

Four X-ray structures of HPL (UniProt ID: P16233), specifically 1GPL, 1LPA, 1LPB, and 1N8S, were retrieved in PDB format from the RCSB Protein Data Bank (https://www.rcsb.org, accessed on 16 July 2024). The protein structures were prepared using the Protein Preparation Wizard, part of the Schrödinger Suite (Maestro, Schrödinger, LLC, New York, NY, USA, 2024). Water molecules, calcium ions, and other co-crystallized molecules were removed during this preparation. Bond orders were assigned automatically, and hydrogen atoms were added at pH 7.4. The CAVIAR software (version 1.0) [34] was used to confirm the binding cavity in the structure of protein 1LPB. CAVIAR identifies and characterizes putative binding cavities in protein structures, providing scores that indicate the likelihood of each cavity serving as a functional binding site based on parameters such as cavity volume, solvent accessibility, and potential for hydrophobic and hydrophilic interactions. Three potential binding cavities were identified by CAVIAR. Two of these were occupied by octyl beta-D-glucopyranoside, a detergent intended for solubilizing membrane-bound proteins in their native state. The third identified cavity coincided with the pocket occupied by the ligand and was selected for docking calculations. A detailed analysis of this cavity included inspecting its physicochemical properties, such as charge distribution, the presence of critical amino acid residues for ligand binding, and the shape and size of the cavity.

#### 2.6.2. Ligand Preparation

The preparation of the ligands involved the following three steps: preparing the co-crystallized ligand extracted from the X-ray structure 1LPB, assembling a dataset of 51 compounds from the ChEMBL database with previously determined IC_50_ values, and preparing the flavonoid library used in this study. The 3D structure of the co-crystallized ligand and the dataset of HPL inhibitors retrieved from ChEMBL were prepared using the LigPrep tool within the Maestro Schrödinger interface (LigPrep, Schrödinger, LLC, New York, NY, USA, 2024). This preparation accounted for all relevant tautomeric and ionization states, ring conformations, and stereoisomers at pH 7.4, and for enumerating the stereoisomers for structures with non-explicit chirality at stereocenters. The LigPrep tool was utilized to construct a library containing 48 flavonoids and resveratrol. Molecular conformations were generated using the OPLS4 force field, and protonation states were adjusted to a pH of 7.4 using the Epik feature. Stereochemistry was carefully considered during the preparation process. The option “Determine chiralities from 3D structure” was selected, permitting the generation of up to 32 stereoisomers per ligand. LigPrep generated multiple output structures for each input structure using varying protonation states, stereochemical configurations, tautomers, and ring conformations. Only a single structure with the lowest energy per ligand was retained for each ligand, to be used in docking calculations. The correct chirality of the ligands was confirmed for all 48 flavonoids tested in this study.

#### 2.6.3. Molecular Docking Protocol (Validation and Execution)

The evaluation of X-ray structures, various software packages, and scoring functions in reproducing experimental poses through molecular docking was undertaken to validate the docking protocol. Among the four available X-ray structures for PL, UniProtID: P16233 (1GPL, 1LPA, 1LPB, and 1N8S) at the RCSB Protein Data Bank (https://www.rcsb.org/, accessed on 17 November 2024), only the last three structures are complete. Of these, the 1LPB structure (a PL-colipase complex inhibited by a C11 alkyl phosphonate) exhibits the highest experimental resolution of 2.46 Å, and was therefore selected for the docking calculations conducted in this study. The other structures, 1LPA and 1N8S, with resolutions > 3 Å, were excluded.

Self-docking for the 1LPB structure was performed using AutoDock Vina 1.2.0 [35], Glide 1.0 [36], and GNINA 1.0 [37], utilizing scoring functions such as Affinity Score, Glide XP, and CNN Score. In the 1LPB structure, the ligand is covalently bonded to SER152, necessitating the removal of the covalent bond and the adjustment of valences for accurate modeling using non-covalent docking. The best root mean square deviation (RMSD) value was obtained in the self-docking calculations using GNINA 1.0 software, 3.5 Å. This value exceeds the 2 Å typically accepted for validating docking protocols. However, it is essential to consider that this involves reproducing the pose of a covalently bonded ligand in the X-ray structure, using non-covalent docking calculations. Non-covalent redocking is vital, as it is used for the compounds tested in this study, allowing us to verify that the docked X-ray ligand nearly wholly overlaps with the experimental pose. The resultant RMSD primarily results from a greater displacement from SER152 and a slight compound rotation due to the absence of a covalent bond. We also performed covalent self-docking for the 1LPB structure using GNINA 1.0 as an extra validation. The RMSD in this case was 2 Å (only the terminal part of the flexible aliphatic chain of orlistat was slightly shifted).

To confirm that the chosen structure and which software (including scoring functions) was capable of reproducing the experimental pose and predicting inhibitory activity in PL, the performance in retrieving actives from a database of compounds with tested inhibitory activity sourced from ChEMBL (release 33, http://www.ebi.ac.uk/chembl, accessed on 17 November 2024) was evaluated, assessing the area under the receiver operating characteristic curve (ROC-AUC) metrics. The protocol validation database consisted of 51 molecules sourced from the ChEMBL 33 database, comprising 12 active and 39 inactive compounds. Compounds with IC_50_ values of less than 10 μM were considered active, while those with IC_50_ values of greater than 10 μM were deemed inactive. This cutoff was chosen to ensure a more significant number of active molecules in the dataset, increasing the sensitivity of the screening and ensuring that most compounds exhibiting relevant biological activity were correctly classified as active, even if they showed moderate activity. This docking protocol validation procedure was crucial to assess the software’s performance and ability to replicate experimental results. Enrichment curves were employed to evaluate the effectiveness of these ranking algorithms.

#### 2.6.4. Visualization of Docking Poses and Protein–Ligand Interaction Analysis

To assess protein–ligand interactions, the top docking poses underwent residue contact detection, using the docker implementation of the Protein–Ligand Interaction Profiler (PLIP) [38]. This analysis included identifying hydrogen bonds, hydrophobic interactions, π-π stacking, and other relevant contacts between the protein and ligand.

Images of the compounds and the corresponding PDB structures were generated using PyMOL v.1.8.4.0 (http://www.pymol.org). These visualizations helped in understanding the spatial arrangement and interaction sites of the ligands within the binding pocket.

Data visualizations were created using the Seaborn library, providing clear and informative graphical representations of the interaction data. Compound structures were processed and analyzed using BioPandas 0.5.1 [39], OpenBabel (http://www.openbabel.org), and RDKit 2023.09.1 (http://www.rdkit.org). The docking results were managed and analyzed through a workflow in Jupyter Notebook, facilitating an organized and reproducible analysis pipeline.

## 3. Results

### 3.1. Porcine Pancreatic Lipase—In Vitro Inhibition

The tested flavonoids were divided into seven groups (A–G) based on their substitution pattern (Table 2). The flavonoids under study include flavones (group A, B, D, E, and G), flavonols (group C) and flavan-3-ols (group F), allowing for an evaluation of the influence of different hydroxy, glycosyl, rutinosyl, galloyl, and extended alkyl groups on the inhibitory effect.

Flavones in group A (**1** to **8**) exhibit differences in substitution patterns at C-5 of the A-ring, as well as at the C-3′ and C-4′ of the B-ring. Flavones in group B (**9** to **16**) have a 7-hydroxy group on the A-ring and different hydroxy substituents at C-5 of the A-ring, as well as at the C-3′ and C-4′ of the B-ring. Also in group B, isoflavone **17** has hydroxy groups at the C-5 and C-7 of the A-ring and C-4′ of the C-ring. Flavonols (flavones with a 3-hydroxy group on the C-ring) in group C (**18** to **22**) have different hydroxy substituents at C-7 of the A-ring as well as at the C-3′ and C-4′ of the B-ring. Flavones from group D (**23** to **35**) have a 5-hydroxy group on the A-ring as well as hydroxy, glucosyl, rutinosyl, and/or extended alkyl substituents at C-7 of the A-ring, C-3 of the C-ring, and C-3′ and C-4′ of the B-ring. In group E (**36** to **38**), flavones have a pyrogallol group at the C-2′, C-3′, and C-4′ of the B-ring, different hydroxy substituents at C-5- and C-7 of the A-ring, and C-3 of the C-ring. Flavan-3-ols (flavones with a 3-hydroxy group on the C-ring) from group F (**39** to **41**) have hydroxy groups at the C-5 and C-7, and C-2′ and C-3′ of the A-ring and C-ring, respectively, as well as different hydroxy and/or galloyl substituents at the C-4′ of the B-ring and C-3 of the C-ring. Finally, the flavones from group G (**42** to **48**) have a 7-hydroxy group on the A-ring and different hydroxy and/or glucosyl substituents at the C-5 and C-8 of the A-ring, C-3′ and C-4′ of the B-ring, and C-3 of the C-ring.

At the maximum concentrations that could be tested (between 25 μM and 100 μM, depending on the compound and its intrinsic fluorescence), no significant inhibitory activities were observed from the flavones in group A.

Among the flavones in group B, compound **16** (luteolin) showed the highest effectiveness, with an IC_50_ value of 32 ± 2 μM, followed by flavonoids **15** (apigenin) and **14**, with IC_50_ values of 41 ± 1 μM and 73 ± 7 μM, respectively. Additionally, due to their intrinsic fluorescence, flavones **11** and **12** were not able to be tested, and the isoflavone tested within this group (**17**) did not show significant inhibitory activity at the highest tested concentration.

In group C, almost all flavonols could not be tested due to their intrinsic fluorescence, except for flavonol **18**, which at the maximum concentration that could be tested (50 μM), did not show significant inhibitory activity.

Flavones from group D were the most active compounds when compared with the flavonoids from the other groups. The most effective were flavones **30**, **29,** and **28**, with IC_50_ values of 7.3 ± 0.6 μM, 10.1 ± 0.4 μM, and 13 ± 1 μM, respectively, followed by flavone **27** (quercetin) and **24** (kaempferol), with IC_50_ values of 27 ± 2 μM and 79 ± 6 μM, respectively. Flavones with glucosyl and rutinosyl groups [**25** (astragalin), **26** (populnin), **31** and **32** (rutin), **33** (quercimeritrin), **34** (spiraeoside), and **35**] did not show significant inhibitory activity at the maximum concentration that could be tested (50 or 100 μM).

Flavones **38** (myricetin) and **36** exhibited the highest inhibitory activity among the compounds from group E, with IC_50_ values of 16 ± 1 μM and 20 ± 3 μM, respectively. However, flavone **37** (robinetin) could not be tested due to its intrinsic fluorescence, similar to some flavonoids from other groups.

In group F, the only flavan-3-ol that showed inhibitory activity was the compound **41** [(**–**)-epigallocatechin gallate, EGCG], with an IC_50_ value of 32 ± 4 μM.

From the studied flavones from group G, only one, **42** (norwogonin), exhibited inhibitory activity, with an IC_50_ value of 98 ± 9 μM. On the other hand, flavone **48** (gossypin), which contains a glucosyl group, did not show significant inhibitory activity even at the highest concentration that could be tested (12.5 μM), as previously observed with flavonoids with similar substituents.

**Figure 2:**
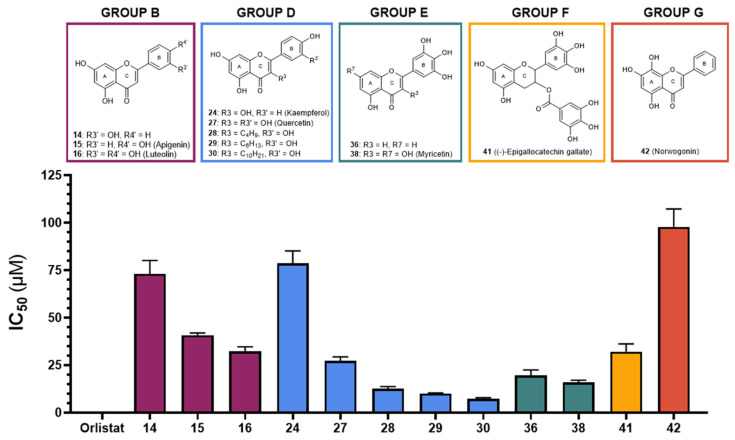
Graphical representation of IC_50_ values of active flavonoids and orlistat as inhibitors of porcine pancreatic lipase (μM, mean ± SEM, n ≥ 3).

Orlistat, which was used as a positive control, exhibited an IC_50_ of 0.0043 ± 0.0003 μM, which was statistically different from the IC_50_ values observed for the flavonoids under study. Figure 2 summarizes the results for all active flavonoids against PPL.

### 3.2. Human Pancreatic Lipase—In Vitro Inhibition

After the analysis of the results obtained using PPL, the inhibitory effects of the flavonoids under study were also tested using HPL as the enzyme source. As seen in Table 2, from the tested flavonoids, at the maximum concentrations that could be tested, no significant inhibitory activities were observed from the flavones in group A.

In group B, flavone **14** showed the highest effectiveness, with an IC_50_ value of 54 ± 5 μM, followed by flavone **15** (apigenin), with an IC_50_ value of 75 ± 2 μM. The only isoflavone tested in this study (**17**) also did not show significant inhibitory activity against HPL at the highest tested concentration.

From group C, the only flavonol that could be tested (**18**) did not show significant inhibitory activity, while in group D, the only flavones that were effective were flavones **30**, **29,** and **28**, with IC_50_ values of 3.2 ± 0.1 μM, 16.4 ± 0.7 μM, and 42 ± 2 μM, respectively. Once again, flavones with glucosyl and rutinosyl groups [**25** (astragalin), **26** (populnin), **31** and **32** (rutin), **33** (quercimeritrin), **34** (spiraeoside), and **35**] did not show significant inhibitory activity against HPL at the maximum concentration that could be tested.

Flavones **36** and **38** (myricetin), the compounds that could be tested from group E, exhibited inhibitory activity against HPL, with IC_50_ values of 7 ± 1 μM and 25 ± 2 μM, respectively.

While in group F none of the flavan-3-ols that were tested exhibited inhibitory activity, in group F, only the flavone **42** (norwogonin) exhibited inhibitory activity against HPL, with an IC_50_ value of 79 ± 3 μM. As noted previously, with flavonoids with similar substituents, the flavone with a glucosyl group (**48,** gossypin) did not show significant inhibitory activity.

**Figure 3:**
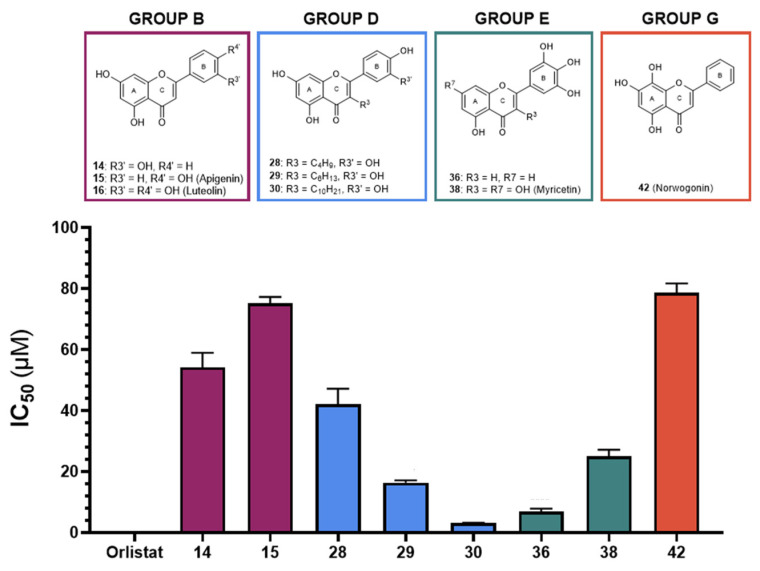
Graphical representation of IC_50_ values of active flavonoids and orlistat as inhibitors of human pancreatic lipase (μM, mean ± SEM, n ≥ 3).

Similarly to HPL, orlistat was used as a positive control, with an IC_50_ value of 0.00068 ± 0.00003 μM, also showing statistically significant differences from the IC_50_ values observed for the flavonoids under study. Figure 3 summarizes the results for all active flavonoids against HPL.

### 3.3. Porcine Pancreatic Lipase—Inhibitory Kinetics

Compounds **27** (quercetin), **30**, **38** (myricetin), and **41** (EGCG) were also selected for the calculation of their type of inhibition against PPL. As described in previous studies, **orlistat** is an irreversible PL inhibitor [40]. For this reason, type of inhibition was not calculated for this compound.

By comparing the sum of squared errors across the different enzyme inhibition models and using the extra sum-of-squares F test and A/C test for models with varying parameters, the optimal model (without inhibition, competitive inhibition, noncompetitive inhibition, uncompetitive inhibitions, or mixed inhibition) was selected. As the first criterium, the model with the lowest sum of squared error values was considered the best kinetic model. To compare models, the extra sum-of-squares F test was used at the desired level of probability (*f* 0.95). Whenever the -*f* value was greater than 0, the more complex model was applied. The Akaike test was also used to distinguish between different enzyme inhibition models; the model with the lower corrected A/C (A/Cc) score was more likely to be the corrected model (see Supplementary Material).

Thus, based on the results presented in Figure 4 and the Supplementary Material, it can be inferred that flavone **27** (quercetin), flavone **30**, and flavan-3-ol **41** (EGCG) are PPL competitive inhibitors (see Supplementary Material, Figures S1–S8 and S13–S16), while flavone **38** (myricetin) is a mixed-type inhibitor (see Supplementary Material Figures S9–S12).

After the determination of the best model, it was possible to calculate the kinetic constant values (V_max_, K_m_, K_ic_, and/or K_iu_, depending on the enzyme inhibition model, Table 3).

Based on the K_ic_ values, the tested flavonoids exhibited the following order for the PPL enzyme: **41** (EGCG) > **27** (quercetin) > **38** (myricetin) > **30**.

### 3.4. Human Pancreatic Lipase—Inhibitory Kinetics

**Figure 5:**
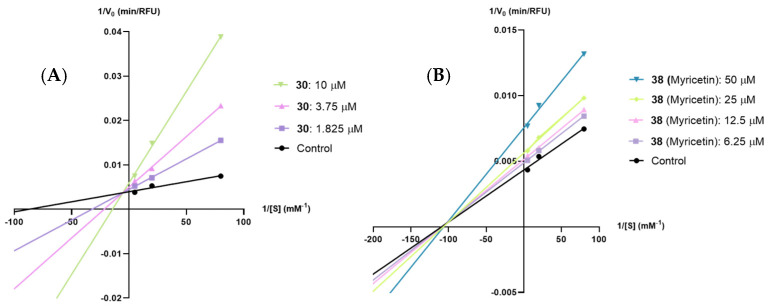
Lineweaver–Burk plots of the 4-methylumbeliferone (4-MU) released in the presence of various concentrations of (**A**) flavone **30** and (**B**) flavone **38** (myricetin) for human pancreatic lipase. Each line was obtained with data from at least three independent experiments.

From the compounds selected for the calculation of their type of inhibitions against PPL, the only compounds that could be tested against HPL were compounds **30** and **38** (myricetin), as the remaining compounds lost their inhibitory effectiveness. Based on the results presented in Figure 5 and the Supplementary Material, it is possible to conclude that flavones **30** and **38** (myricetin) are HPL mixed-type and noncompetitive inhibitors, respectively, (see Supplementary Material, Figures S17–S24).

As for the kinetic constant values, as seen in Table 4, based on the *K*_ic_ values, the tested flavonoids exhibited the following order: **38** (myricetin) > **30**.

### 3.5. Human Pancreatic Lipase—Computational Studies

**Figure 6:**
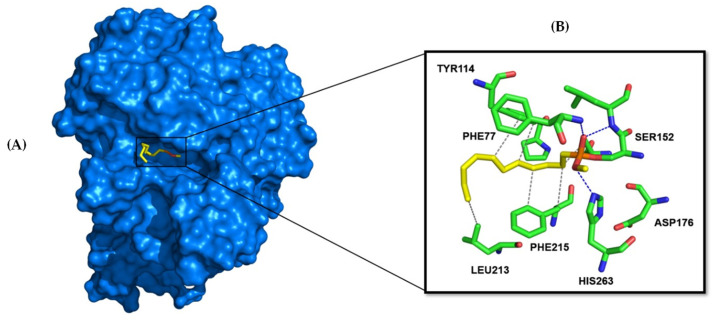
X-ray structure of the human pancreatic lipase (HPL) protein (PDB ID 1LPB). (**A**) The HPL protein is depicted as a blue surface, with its co-crystallized ligand (C-11 alkyl phosphonate shown as yellow sticks) located within the catalytic cavity. (**B**) Close-up view of the catalytic site cavity, with the pocket’s catalytic residues highlighted in green and the C-11 alkyl phosphonate shown as yellow sticks.

HPL consists of 465 amino acids and features a catalytic triad near the N-terminus, comprising SER152, HIS263, and ASP176 (Figure 6). Typically, the conformation of the lipase changes results in the ’lid’ opening and exposing the hydrophobic region of the active site, thereby facilitating substrate binding and catalysis. As previously mentioned, orlistat, a potent and specific inhibitor of PL, is the only compound currently used clinically to manage obesity. It exerts its pharmacologic activity by forming a covalent bond with the active site serine of gastric and pancreatic lipases in the gastrointestinal tract, preventing these enzymes from hydrolyzing dietary fats (in the form of triacylglycerides) into absorbable free fatty acids and glycerol [41]. To elucidate the essential structural requirements for HPL inhibition and to explore the structure–activity relationship, a library of 48 structurally related flavonoids, along with orlistat (Table 2), was docked into the active site of HPL (PDB ID: 1LPB, Figure 6, http://www.pymol.org). Molecular docking was chosen for this study because it demonstrates good performance in evaluating the affinity of a compound for a specific receptor. This method allows for the prediction of binding modes and interaction energies, facilitating the identification of potential drug candidates and the optimization of lead compounds by providing insights into the molecular interactions at the atomic level.

The initial stage of this study involved validating the most suitable docking protocol for evaluating the affinity of compounds in the flavonoid library as HPL inhibitors. Four available X-ray structures for HPL (1GPL, 1LPA, 1LPB, and 1N8S) were identified during this stage. Among these, only the 1LPB structure had a complete structure, a resolution < 2.5 Å, and a co-crystallized ligand, C-11 alkyl phosphonate (Figure 6). To identify and confirm the binding pocket of HPL for docking calculations, we employed the CAVIAR 1.0 software. In the absence of suitable structures for cross-docking analysis, only self-docking was performed, using three different docking software programs (GLIDE, AutoDock Vina, and GNINA). The non-covalent docking poses were then compared with the crystallographic pose, to assess if our protocol could reproduce the experimental data. The ligand poses obtained from self-docking using GLIDE, AutoDock Vina, and GNINA scoring metrics yielded RMSD (root mean square deviation) values of 4.89 Å, 3.62 Å, and 3.50 Å, respectively. As a result, GNINA was considered the most suitable software for docking studies, as it achieved the lowest RMSD value, indicating its superior ability to reproduce the experimental data.

**Figure 7:**
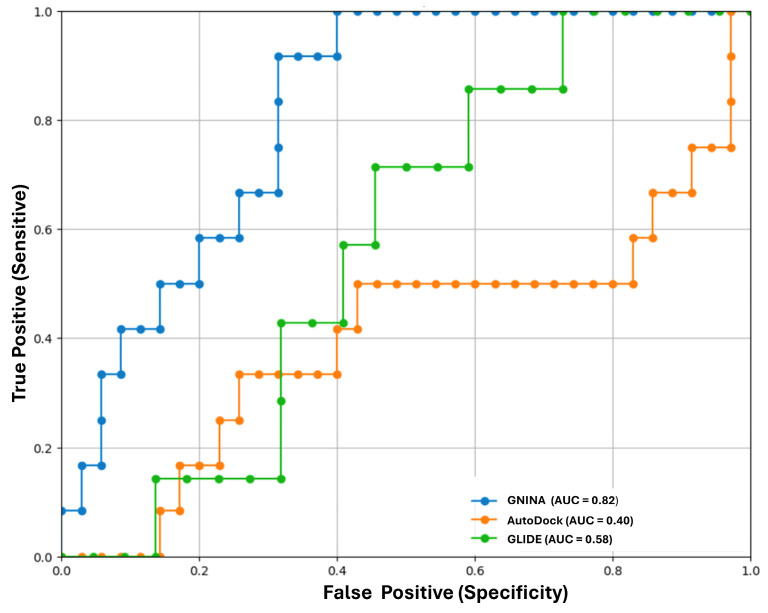
Receiver operating characteristic (ROC) curves and area under the curve (AUC) metrics for retrospective evaluation of docking performance of ChEMBL dataset against human pancreatic lipase protein. This figure illustrates the ROC curves and AUC metrics derived from docking studies involving 51 compounds (12 actives and 39 inactives) from the ChEMBL database (release 33), using AutoDock Vina, Glide, and GNINA software.

To further refine the validation process, receiver operating characteristic (ROC) curves were generated for each software/scoring function, plotting the true positive rate against the false positive rate (Figure 7). The performance metric was the area under the curve (AUC), with AUC = 1 representing a perfect classifier and AUC = 0.5 being no better than chance. The screening validation dataset included 51 molecules from the ChEMBL 33 database [42], consisting of 12 actives and 39 inactives. This systematic approach allowed for a comprehensive evaluation of the effectiveness of the proposed validation method, ensuring a technically informed analysis for software selection.

The ROC curves for all three software packages indicated that GNINA exhibited superior docking performance. GNINA achieved an AUC of 0.82, significantly surpassing the results obtained by Glide and AutoDock Vina, which had AUC values of 0.58 and 0.40, respectively. Both the RMSD and AUC indicate that GNINA is the most effective method for conducting molecular docking calculations for the 48 flavonoids.

**Figure 8:**
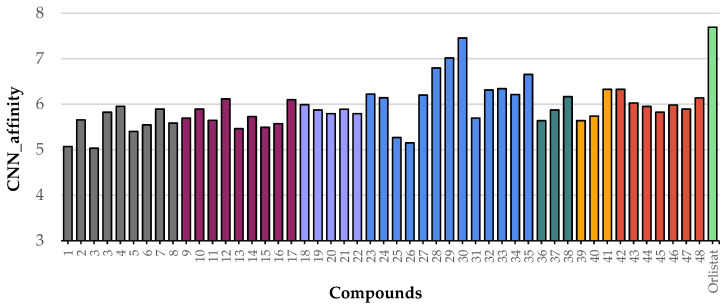
Graphical representation of GNINA docking scores (CNN Affinity) for the interaction of the flavonoid library (flavonoids **1** to **48**) and orlistat with the human pancreatic lipase protein. Compounds from Group A are represented in grey, Group B in plum, Group C in lilac, Group D in blue, Group E in dark green, Group F in yellow, Group G in red, and orlistat in light green.

After validating the docking protocol, the library of 48 flavonoids (Groups A to G) was docked into the active site of HPL. For comparison, molecular docking studies were also performed with the positive control, orlistat. The GNINA (CNN Affinity) scores for the best docking poses of these flavonoids ranged from 5.034 for compound **5** to 7.455 for flavone **30** (Figure 8). Under the same conditions, orlistat achieved a CNN Affinity score of 7.693 (Figure 8).

**Figure 9:**
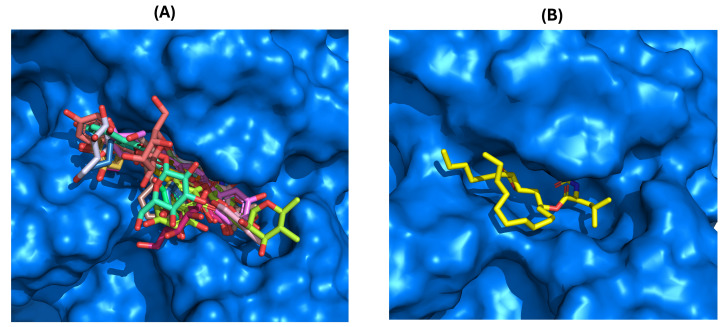
(**A**) Representative selection of top-scoring docked poses (non-covalent docking) of flavonoids from the dataset within the human pancreatic lipase (HPL) binding pocket. (**B**) Top docking pose of orlistat (in yellow) in the HPL binding pocket, with the binding pocket surface highlighted in blue. Figures were generated using Schrödinger PyMOL v.1.8.4.0.

The docking calculations revealed that all compounds are positioned in the same pocket region as the ligand present in the X-ray structure and the inhibitor orlistat. This positioning suggests potential interactions with key residues, specifically PHE77, TYR114, and PHE215, and the catalytic residues SER152 and HIS263 (Figure 9A). These residues are among the eight interactions established by the crystallographic ligand. All active compounds from the tested library exhibit interaction profiles that are, in some ways, similar to orlistat (Figure 9B). This includes the active flavones **14** and **15** (apigenin, Group B); **28**, **29**, and **30** (Group D); **36** and **38** (myricetin, Group E); and **42** (norwogonin, Group G).

**Figure 10:**
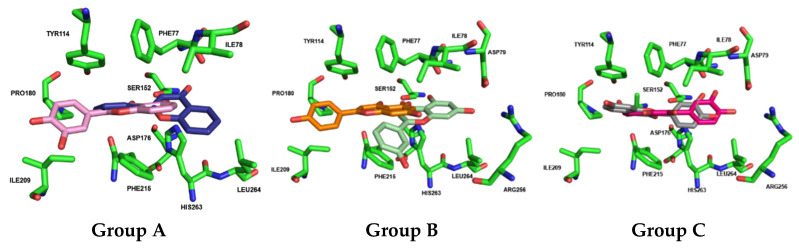
Docked poses (non-covalent docking) and interactions of flavonoids from Group A (compounds **1** in blue and **8** in pink), Group B (compounds **14** in orange and **15** (apigenin) in light green), and Group C (compounds **18** in bright pink and **22** (fisetin) in gray) against human pancreatic lipase (pocket residues in green). Figures were generated using Schrödinger PyMOL v.1.8.4.0.

Docking calculations for the compounds in Group A (flavones **1** to **8**) revealed diverse placement possibilities within the binding pocket. While some of these compounds position the A-ring and C-ring similarly to flavone **30** (the most active in this series), the absence of hydroxy groups and side chains significantly limits their interactions. Flavones **5**, **6**, **7**, and **8** exhibit perfect overlap in the orientation of the A-ring and C-ring. In contrast, flavones **2**, **3**, and **4** invert their orientation, positioning the B-ring deeper within the pocket, with flavone **3** showing a discernible shift by binding to a more external part of the pocket (Figure 10).

Similarly, in Group B (flavones **9** to isoflavone **17**), various orientations within the HPL binding pocket were observed. While flavones **10** and **12** position the B-ring towards the deeper part of the pocket, flavones **15** (apigenin) and **16** (luteolin) exhibit overlapping poses in the pocket, similar to flavone **14** and isoflavone **17** (genistein). However, differences in the substitution position (*meta* and *para*) of the B-ring cause orientation variations in the final pose (Figure 10).

In Group C (flavonols **18** to **22**), the following two distinct modes of interaction were observed: flavonols **18**, **20**, and **22** (fisetin) direct the B-ring towards the deeper part of the pocket, whereas flavonols **19** and **21** position the A-ring and C-ring in that region (Figure 10).

**Figure 11:**
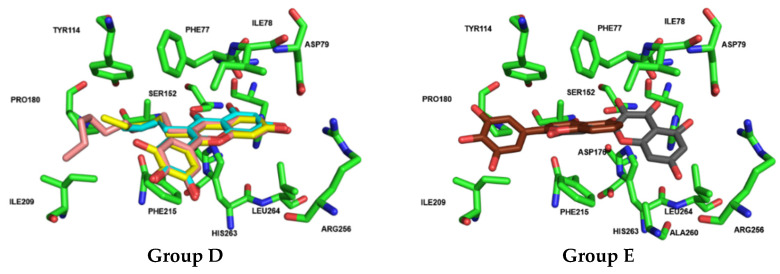
Top-scoring docked poses (non-covalent docking) and interactions of flavonoids from Group D (flavones **28** in yellow, **29** in cyan, and **30** in salmon), and Group E (flavones **36** in brown and **38** in dark gray) against human pancreatic lipase (pocket residues in green). Figures were generated using Schrödinger PyMOL v.1.8.4.0.

Docking results for Group D (flavones **23** to **35**) showed nearly identical poses for flavones **23** (galangin), **24** (kaempferol), and **27** (quercetin), with the A-ring and C-ring oriented similarly towards the deeper part of the binding pocket. A similar pattern was observed for flavones **28**, **29**, and **30**, where the A-ring and C-ring were stabilized by hydrogen bonds formed between the hydroxy groups on the A-ring and the amino acid residues HIS151, HIS263, and ARG256. Additionally, hydrophobic π–π interactions were observed between the B-ring and PHE215. Substituents at the *ortho* position of the C-ring (R3: C_4_H_9_, C_6_H_13_, and C_10_H_21_) enhanced pocket occupancy and engaged in interactions with critical residues such as TYR114, PRO180, ILE209, and PHE215, which are essential for inhibitory activity. Both orlistat and the crystallographic ligand also occupy this pocket region, and either interact with or are proximal to these residues (Figure 11). These compounds were among the most active in the in vitro assays of the inhibitory effects of flavonoids on HPL. The docking results confirm the experimental observation that longer chains in this position increase the inhibitory activity of HPL, and show that this increase is due to the enhanced interactions established by the chain. For example, in this group, only compound **30** establishes interactions with ILE209 at the end of the aliphatic chain. An interaction that our docking calculations show also exists in compound **36** (the second most active of all the compounds tested in this study).

The docking calculations further demonstrate that active flavones **36** and **38** (myricetin) from Group E are well positioned within the binding pocket, with both orienting the A-ring and C-ring towards its deeper region. Flavone **38** (myricetin) penetrates deeper into the binding cavity, enabling it to form hydrogen bonds with PHE77, ARG256, HIS263, and HIS264. Although flavone **36** partially overlaps with flavone **38** (myricetin), it shifts approximately 5 Å towards the protein interface within the pocket. Despite this displacement, flavone **36** successfully establishes π–π and π–cation interactions with PHE77 and PHE215 (Figure 11). Although flavone **36** did not achieve one of the highest docking scores, potentially due to its smaller size, its orientation within the active site of HPL demonstrates that flavone **36** forms a key interaction with ILE209. This interaction is also noted in compound **30,** and is present in the interaction profile of orlistat with the enzyme. Such findings may clarify the effective experimental inhibition observed for these compounds.

**Figure 12:**
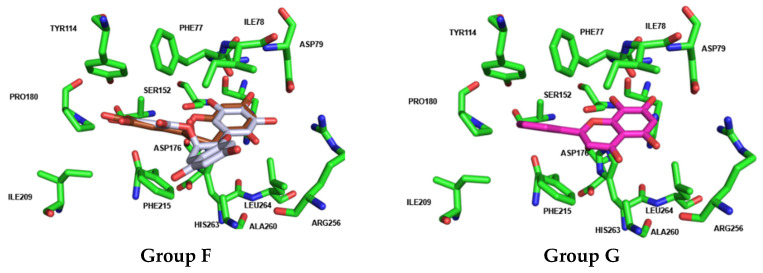
Docked poses (non-covalent docking) and interactions of compounds from Group F (flavan-3-ol **40** in brown and **41** in dark gray) and Group G (flavan-3-ol **42** in bright pink) with human pancreatic lipase (pocket residues in green). Figures were generated using Schrödinger PyMOL v.1.8.4.0.

Docking calculations for Group F (flavan-3-ols **39** to **41**) show that flavan-3-ols **39** [(–)-epicatechin] and **40** [(–)-epigallocatechin] assume a fully overlapped pose in the binding pocket of HPL, closely resembling the pose adopted by flavan-3-ol **42** (norwogonin) from Group G. In contrast, flavan-3-ol **41** (EGCG) exhibits a distinctly different pose, with the hydroxy groups on the A-ring extending outward from the pocket (Figure 12).

Docking calculations for flavones **42** (norwogonin), **43**, **44**, and **46** (herbacetin) from Group G revealed that these compounds adopt fully overlapping poses within the binding pocket. In this orientation, the aromatic moieties of the A-ring and C-ring engage in π–cation interactions with HIS263, HIS264, and PHE215. Additionally, the hydroxy substituents at C-7 and C-8 of the A-ring form hydrogen bonds with the amino acids PHE77, HIS263, and the catalytic SER152. Moreover, the B-ring establishes π–π and π–cation interactions with the amino acids PHE215, PRO180, and TYR114 within the pocket. Although flavone **47** (gossypetin) is located in the same region, it exhibits a slight rotation, a pose also observed for flavone **48** (gossypin). The substituent at C-8 of the A-ring (Glu) does not significantly alter the pose or increase activity, as it occupies a more exposed region of the pocket. The most active compound within this group is flavone **42** (Norwogonin), which is notably the only compound in the group that interacts with PRO180 (Figure 12).

**Figure 13:**
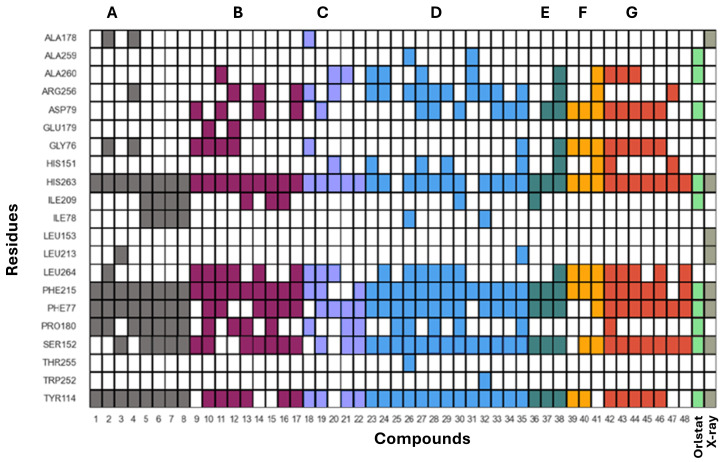
Binary heat map that illustrates the presence or absence of interactions between flavonoids and specific amino acids from human pancreatic lipase (HPL) obtained from docked poses. Each cell in the heat map indicates whether a particular compound (listed on the *x*-axis) interacts with a specific amino acid residue in HPL (listed on the *y*-axis). A dark-colored cell denotes that an interaction is formed, while a white cell indicates no interaction. Compounds from Group A are represented in grey, Group B in plum, Group C in lilac, Group D in blue, Group E in dark green, Group F in yellow, Group G in red, and orlistat in light green.

In this study, we also compared the interaction patterns of the 48 flavonoids with orlistat and the X-ray ligand pose to identify key structural features and interactions (Figure 13). These features, combined with the docking scores, help to elucidate the observed activities or their absence, providing valuable insights into the molecular basis of the inhibitor’s effectiveness or lack thereof.

**Figure 14:**
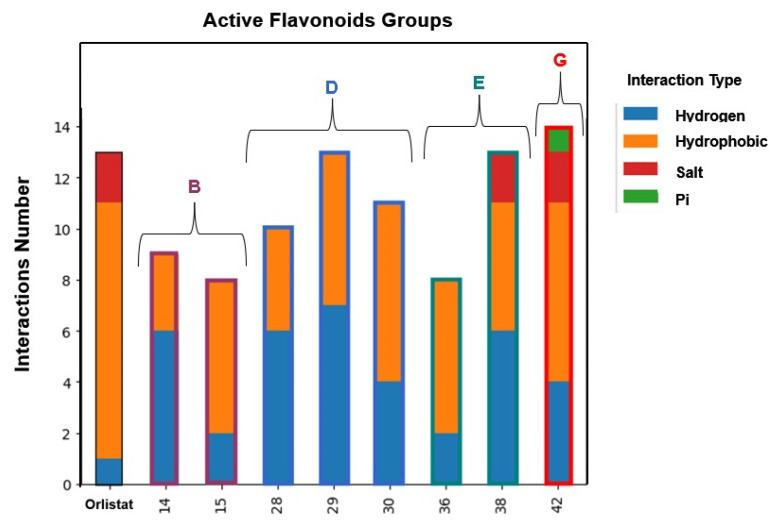
Comparison of interaction types established by orlistat and the most active flavonoids belonging to Groups B, D, E, and G with human pancreatic lipase pocket residues.

By examining the amino acids interacting with the tested flavonoids, the types and numbers of interactions established by the most active compounds against HPL were determined (Figure 14). It was observed that all flavonoids established eight or more interactions. Notably, the most active compound in this study, flavone **30**, established four hydrogen bond interactions and seven hydrophobic interactions.

The docking scores for the flavonoid series, as well as for orlistat with HPL, effectively reflect the strength and nature of interactions between these compounds and the target protein. The results demonstrate a strong correlation between the GNINA docking scores and the inhibitory activity against HPL, as detailed in Table 5 for active flavonoids and Table S1 for all the studied flavonoids (see Supplementary Material, Table S1).

## 4. Discussion

Given the alarming worldwide increase in obesity in both adults and children reported in recent data, the search for safe and effective drugs leading to weight loss is gaining special attention. Considering the importance of PL in lipid absorption, its inhibition has been studied as an effective approach to weight loss. However, hitherto, orlistat is the only PL inhibitor approved as an anti-obesity drug. Despite being quite effective in weight loss, its unpleasant side effects often make it an undesirable option and may lead to drug discontinuation by patients. Therefore, it is crucial to find safer alternatives to fight the global epidemic of obesity and its associated complications [43,44].

In this work, a panel of 48 flavonoids were tested as potential PL inhibitors using PPL and HPL. The flavonoids were divided into seven groups (A–G) according to the number and the position of different substituents (hydroxy, glycosyl, rutinosyl, galloyl, and extended alkyl groups), to establish a structure–activity relationship. Besides the observed inhibitory activity against PPL and HPL, kinetic analysis and different inhibitory models were also tested for flavonoids that showed the highest effectiveness in the inhibition of PL catalytic activity.

The results of this study demonstrate that several of the studied flavonoids are effective inhibitors of PPL and HPL, and that PL catalytic activity inhibition is dependent on the substituents and their position within the flavonoid scaffold. Among the studied compounds, flavone **30** was the most effective against both enzymes (PPL and HPL) besides orlistat. These findings were corroborated by our docking calculations for HPL.

Before the inhibition assays, a preliminary study was conducted to investigate the potential interference of the flavonoids under study with the assay conditions, due to their possible intrinsic fluorescence and/or absorption in the wavelengths used in the assays. As a result, the maximum concentration for some flavonoids was below 100 μM, while some flavonoids [**11**, **12**, **19–22** (fisetin), and **37** (robinetin)] could not be tested.

**Figure 15:**
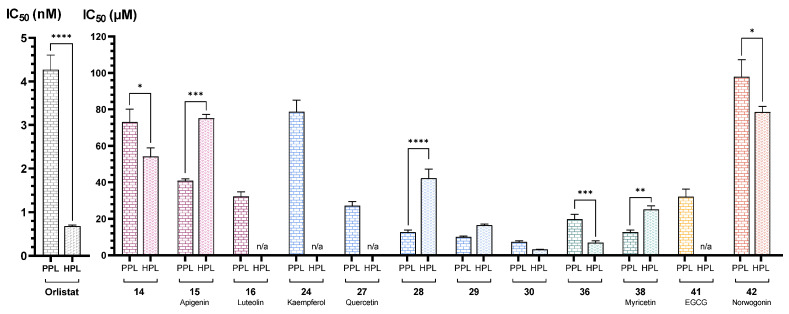
Graphical representation of IC_50_ values of active flavonoids and orlistat for porcine and human pancreatic lipase (μM and nM, mean ± SEM, n ≥ 3). *, **, ***, and **** indicate that IC_50_ values of the compounds for porcine and human pancreatic lipase are statistically different (*p* < 0.05, *p* < 0.01, *p* < 0.001, and *p* < 0.0001, respectively). (–)-Epigallocatechin gallate (EGCG); not applicable (n/a); porcine pancreatic lipase (PPL); and human pancreatic lipase (HPL).

Considering the positive control, a comparison between PPL and HPL revealed that orlistat, while effective in the catalytic inhibition of both enzymes, exhibited greater inhibitory potency with statistically significant difference in the human enzyme (Figure 15). This finding suggests that, despite the enzyme homology, different results could be achieved for the compounds under study depending on the enzyme source.

Regarding group A, the tested flavones (**1**–**8**) did not exhibit significant inhibitory activity against either PPL or HPL, even at the highest tested concentrations. This demonstrates that the catechol in the B-ring (3′ and 4′ positions), along with the hydroxy group at C-5 of the A-ring, are not sufficient to affect the catalytic activity of both enzymes. Our docking calculations reveal that flavones **2**, **3**, and **4**, which lack a hydroxy group at C-5 of the A-ring, exhibit an inverted pose compared to flavone **30**. These compounds tend to orient the B-ring towards the deeper part of the pocket, resulting in a 180º rotation of the entire molecule within the pocket. This orientation limits essential interactions with residues such as SER152, ARG256, and HIS263. In contrast, flavones **5** to **8** maintain the same orientation as flavone **30**, but they are displaced by 4 Ǻ towards the more superficial part of the pocket. This displacement distances them from the catalytic residues. The presence of the hydroxy group at C-5 of the A-ring enables the formation of a hydrogen bond with HIS263, which stabilizes the compound’s pose and prevents it from penetrating more deeply into the HPL pocket. This stabilization explains the shallower distancing of these compounds within the pocket.

In what concerns group B, two flavones [**14** and **15** (apigenin)] demonstrated effectiveness as PPL and HPL inhibitors, while flavone **16** (luteolin) was only effective in PPL catalytic activity inhibition. The inhibitory effect on both PPL and HPL appears to be influenced by the presence of hydroxy groups at C-5 and C-7 of the A-ring. However, inhibition is only observed when hydroxy groups are added to the B-ring at the C-3′ and/or C-4′.

Concerning PPL, the most active flavone was **16** (luteolin, IC_50_ = 32 ± 2 μM), with a catechol group at the B-ring. However, there was no significant difference in the IC_50_ value of this flavone when compared with flavone **15** (apigenin, IC_50_ = 41 ± 1 μM), which only has one hydroxy group at C-4′ of the B-ring. In contrast, flavone **14** (IC_50_ = 73 ± 7 μM), with one hydroxy group at C-3′ of the B-ring, showed a significant difference in the IC_50_ value when compared to flavones **15** (apigenin) and **16** (luteolin). Therefore, the 4-hydroxy group on the B-ring appears to be the substitution that makes the difference in PPL catalytic activity inhibition.

On the other hand, regarding HPL, the most active flavone was **14** (IC_50_ = 54 ± 5 μM), with a slight difference in the IC_50_ value when compared to flavone **15** (apigenin), which showed that, in opposition to PPL, the 3′-hydroxy group on the B-ring seems to have a greater impact on HPL catalytic inhibition. Interestingly, when comparing the results for both enzymes, the most active flavone for PPL (**16**, luteolin), with a catechol group in the B-ring, lost its inhibitory effect against HPL, and the inhibitory potency of the compounds did not show a consistent trend, as the IC_50_ value either increased or decreased depending on the substitution (Figure 15). In the docking poses obtained for flavone **14**, the most active in Group B, the three rings of this compound are completely superimposed with those of flavone **30**. This overlapping pose establishes interactions with SER152, ARG256, and HIS263, which may explain its activity. However, the absence of the C_10_H_21_ chain prevents it from interacting as effectively with the amino acids at the left entrance of the pocket, such as PRO180, leaving this region highly accessible. Although flavone **15** (apigenin) has the same ring orientation, it is positioned in a more external region, with the B-ring oriented exactly where flavone **30** positions the C_10_H_21_ chain. This difference in poses is due to the hydroxy group at C-3′ of the B-ring, which forms a hydrogen bond with ALA259, unlike the substitution at C-4′, where this interaction does not occur. Instead, it prefers to establish an interaction between the carbonyl group of the C-ring and SER152. Comparing the position of flavone **16** (luteolin, with hydroxy groups at C-3′ and C-4′ of the B-ring) with the two previous compounds suggests that the presence of an aliphatic chain at C-3 is essential for the compounds to occupy the entire pocket and inhibit HPL catalytic activity.

Still within group B, the use of isoflavone **17** (genistein, IC_50_ < 30%^100 μM^), a homologue of flavone **15** (apigenin), did not promote the inhibition of any of the enzymes, suggesting that this type of compound might not be the best option. In isoflavone **17** (genistein), the different substitution position of the B-ring causes it to shift to a different part of the HPL pocket, resulting in a slight rotation that leads to the loss of interactions with ARG256 and ALA259. This loss of critical interactions likely contributes to its lack of inhibitory activity.

Concerning group C, the majority of the flavonols could not be tested, due to their intrinsic fluorescence. Thus, regarding PPL and HPL catalytic inhibition, no conclusions can be drawn regarding the effect of the addition of a 3-hydroxy group on the C-ring, in addition to the catechol group in the B-ring. Moreover, these compounds did not achieve favorable scores in the docking calculations. The obtained poses do not indicate an optimal interaction pattern with HPL, suggesting the limited potential of these compounds as effective inhibitors.

In group D, three flavones (**28**, **29,** and **30**) demonstrated effectiveness as PPL and HPL inhibitors, while flavones **24** (kaempferol) and **27** (quercetin) were only effective in PPL catalytic activity inhibition. Comparing flavone **24** (kaempferol, IC_50_ = 79 ± 6 μM) to flavone **15** (apigenin, IC_50_ = 41 ± 1 μM) from group B, which only differ by the addition of a 3-hydroxy group on the C-ring, showed that the addition of this group does not appear to enhance the inhibition of PPL catalytic activity, since there is a statistically significant increase in the IC_50_ value. Notably, a parallel outcome is observed with the human pancreatic lipase (HPL), where the addition of the 3-hydroxy group leads to a discernible loss of inhibitory effectiveness for this enzyme.

Regarding PPL, within group D, when comparing flavone **27** (quercetin, IC_50_ = 27 ± 2 μM) with flavone **24** (kaempferol), the addition of a 3′-hydroxy group on the B-ring and, consequently, the presence of a catechol group in this ring, seems to improve the inhibitory activity of the flavonoid, as there is a significant decrease in the IC_50_ value. This is corroborated by the previous studies of Zhang et al. [31] and Park et al. [45], which also concluded that quercetin is significantly stronger than kaempferol in PPL inhibition. On the other hand, when the comparison is between flavone **27** (quercetin) and flavone **16** (luteolin, IC_50_ = 32 ± 2 μM) from group B, which only differ by the presence of a 3-hydroxy group on the C-ring, the IC_50_ values of these flavonoids are not statistically different. This suggests that, similarly to what was concluded before, the addition of a hydroxy group at C-3 of the C-ring does not appear to enhance PPL catalytic activity inhibition. Considering the human enzyme, the addition of a 3-hydroxy group on the C-ring, similarly to what was observed for PPL, seems not to favor the inhibition of HPL catalytic activity. However, contrary to PPL and similarly to what was concluded previously for this enzyme, the presence of a catechol group in the B-ring does not appear to be enough for HPL catalytic activity inhibition.

Still within group D, the addition of an alkyl chain at the C-3 of the C-ring seems to enhance catalytic activity inhibition in both enzymes. For PPL inhibition, there was a slight decrease in the IC_50_ values as the chain lengths increased, as seen with flavones **28** (IC_50_ = 13 ± 1), **29** (IC_50_ = 10.1 ± 0.4 μM), and **30** (IC_50_ = 7.3 ± 0.6). The lack of significance in the differences among the IC_50_ values of these flavones could suggest that the size of the chain is not a relevant factor in inhibition. However, when comparing these compounds to flavone **27** (quercetin, IC_50_ = 27 ± 2 μM), which only differs by the substitution of the alkyl chain for a hydroxy group, flavone **30** (with the longest chain) was the only one that showed statistically significant differences in the obtained IC_50_ values. On the other hand, for HPL, the decrease in the IC_50_ values was more evident and with significant differences between flavones **28** (IC_50_ = 42 ± 2 μM), **29** (IC_50_ = 16.4 ± 0.7 μM), and **30** (IC_50_ = 3.2 ± 0.1 μM). The importance of the alkyl chain was even more evident within this enzyme when comparing these compounds to flavone **27** (quercetin, IC_50_ < 46% ± 3%^75 μM^), which, in the absence of these substituents, lost its inhibitory effectiveness. It was also interesting to note that, despite the significant difference in the IC_50_ value for both enzymes being observed only for compound **28**, once again, there was no clear trend, as the compounds with sorter chains (**28** and **29**) had an increase in IC_50_ value, while the value for the compound with the longer chain (**30**) slightly decreased (Figure 15). Therefore, to further understand the influence of the size of the alkyl chain in this position, compounds with longer chains should be tested. This is an important finding, since, as far as we know, this was the first time that these flavones were tested as potential PL inhibitors.

To elucidate the enhanced activities of flavones **28** to **30** within Group D against HPL and to clarify the importance of the chain length attached to C-3 of the C-ring, the docking results were analyzed. These three compounds achieved the highest scores in the tested compound library (6.796, 7.015, and 7.455, respectively), approaching the score obtained for orlistat, which was 7.693. Flavones **28**, **29**, and **30** exhibited similar interaction patterns, with the A-ring and C-ring stabilized by hydrogen bonds formed between the hydroxy groups on the A-ring and the amino acid residues HIS151, SER152, HIS263, and ARG256. Additionally, π–π interactions were observed between the B-ring and PHE215. Substituents at the *ortho* position at C-3 of the C-ring (C_4_H_9_, C_6_H_13_, and C_10_H_21_) enhanced pocket occupancy and engaged in interactions with critical residues such as TYR114, PHE77, LEU213, and PHE215, which are essential for inhibitory activity. Both orlistat and the crystallographic ligand also occupy this region of the pocket and either interact with or are proximal to these residues. Furthermore, it is also evident that the presence of bulky substituents at C-7 of the A-ring [flavones **26** (populnin) and **33** (quercimeritrin)] causes the compounds to rotate within the pocket, resulting in the loss of interactions with the essential amino acids SER152, HIS151, and HIS263, which are crucial for inhibiting HPL catalytic activity.

Also in group D, the inhibitory capacity of flavones with glucosyl and rutinosyl groups was tested. However, the addition of these groups in the different positions studied (C-7 of the A-ring, C-4′ of the B-ring, and C-3 of the C-ring) did not favor both PPL and HPL catalytic activity, since all the tested glycosylated flavones lost their inhibitory capacity. For HPL, similar conclusions can be drawn.

Regarding group E, two flavones [**36** and **38** (myricetin)] were found to be effective as PPL and HPL inhibitors. With respect to PPL, a comparison between flavone **36** (IC_50_ = 20 ± 3 μM) and flavone **8** (IC_50_ = 46 ± 2%^50 μM^), which only differ by the presence of a pyrogallol group instead of a catechol group in the B-ring, indicates that the addition of a hydroxy group in this ring has a significant impact on the inhibition of PPL catalytic activity. Flavones **36** and **38** (myricetin, IC_50_ = 16 ± 1 μM) have statistically similar IC_50_ values, which confirms that the addition of a 3-hydroxy of the C-ring and C-7 of the A-ring is not very relevant for PPL catalytic activity inhibition. As for HPL, the importance of the pyrogallol group in the B-ring of flavone **36** (IC_50_ = 7 ± 1 μM) is evident when comparing this compound with flavone **8** (IC_50_ < 30%^50 μM^). Furthermore, as seen in Figure 15, among all the tested compounds, the most substantial decrease in the IC_50_ value between PPL and HPL is observed in flavone **36.** This suggests that this substitution could be a promising alternative for inhibiting HPL catalytic activity. Additionally, similarly to what was concluded before, despite the slight difference between flavone **36** and **38** (myricetin, IC_50_ = 25 ± 2 μM), the addition of a 3-hydroxy of the C-ring and C-7 of the A-ring does not appear to be the most crucial substitution to enhance the inhibition of HPL. Once again, within this group, the absence of a trend in the difference in the IC_50_ value, depending on the enzyme source, was observed (Figure 15).

The docking calculations confirm this assertion. Although flavones **8** and **36** are completely superimposed in their positions within the active site, the additional hydroxy group on the B-ring positions it approximately 4 Å from the hydroxy group of TYR114. This distance could allow for a hydrogen bond mediated by nearby water molecules. Additionally, this hydroxy group is directed towards PRO180 (3.5 Å). Flavone **38** (myricetin), while positioned more internally within the pocket, establishes most of the interactions observed with flavone **36**. However, it is further from external amino acids, such as PRO180.

In group F, only the flavan-3-ol monogallate ester **41** [(–)-epigallocatechin gallate, IC_50_ = 32 ± 4 μM] demonstrated effectiveness as a PPL inhibitor, while for HPL inhibition, none of the compounds were effective. Despite that, the importance of the C2=C3 double bond and the carbonyl group of the C-ring was highly evident in this group for both enzymes. For PPL, when comparing flavone **38** (myricetin, IC_50_ = 16 ± 1 μM) to flavan-3-ol **40** [(–)-epigallocatechin, IC_50_ < 30% ^100 μM^], where it lacks this bond and specific group despite the presence of the pyrogallol group in the B-ring, the flavan-3-ol was unable to inhibit PPL catalytic activity. However, considering flavan-3-ol monogallate ester **41** [EGCG, IC_50_ = 32 ± 4 μM], it was possible to conclude that the absence of the double bond was compensated for by the addition of the 3-galloyl group of the C-ring, which led to the conclusion that esterification of the flavan-3-ol makes a significant difference in PPL catalytic activity inhibition. This is in accordance with previous studies that showed that the presence of a pyrogallol group in the B-ring (C-2′, C-3′, and C-4′) and a galloyl moiety in the C-ring (C-3) improve the inhibitory effects of the flavonoids against this enzyme [46,47,48]. Regarding HPL, the importance of the C2=C3 double bond and the carbonyl group in the C-ring was also evident when comparing flavone **38** (myricetin, IC_50_ = 25 ± 2 μM) to flavan-3-ol **40** [(–)-epigallocatechin, IC_50_ < 30% ^100 μM^]. However, in contrast to PPL, the addition of a 3-galloyl group to the C-ring does not appear to be enough to compensate for the absence of the double bond.

Finally, considering group G, the addition of hydroxy groups at the A-ring appears to slightly enhance the inhibition of both enzymes, but it is not the most crucial factor affecting PPL and HPL catalytic activity. Like what was observed in similar flavonoids, the addition of glycosyl groups does not seem to be the best option for either enzyme. The docking calculations for Group G compounds reveal that flavones **42** (norwogonin)**, 43**, **44**, and **46** (herbacetin) occupy the exact same position in the active site of HPL. These compounds are rotated 180° along the axis of the A-ring and B-ring compared to the most active compound, flavone **30**. An interesting aspect of their pose is that they position the B-ring in the region occupied by the aliphatic chains at C-3 of the C-ring in flavones **28**, **29**, and **30**. The fact that only flavone **42** (norwogonin) exhibits inhibitory activity against HPL underscores the importance of having a hydrophobic moiety in this region.

**Figure 16:**
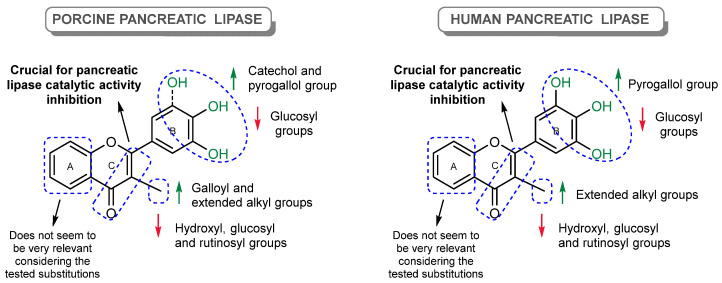
Potential substitution pattern of flavonoids that can contribute to the improvement of or decrease in porcine and human pancreatic lipase catalytic activity inhibition.

Figure 16 provides an overview of the findings regarding the structure–activity relationship and the chemical features that are favorable or unfavorable for the inhibitory effect. To the best of our knowledge, this is the first work that has tested the inhibitory effects of a large panel of flavonoids under the same experimental conditions against the catalytic activity of PPL and HPL, which allowed for the establishment of a more accurate structure–activity relationship.

Regarding both PPL and HPL enzymes, as mentioned before, all the studied compounds were found to be less potent than the positive control (orlistat), as all flavonoids exhibited higher IC_50_ values. Since lower IC_50_ values indicate higher potency and less toxicity of the compound/inhibition, this suggests that orlistat remains a more effective inhibitor. However, it is important to highlight that orlistat’s inhibitory actions are associated with gastrointestinal side effects and the potential malabsorption of fat-soluble vitamins, raising safety concerns. In contrast, as discussed before, flavonoids offer potential advantages. Their low bioavailability may limit systemic toxicity while promoting a localized action in the gastrointestinal tract, which is precisely where PL inhibition is most relevant. While flavonoids demonstrated lower potency than orlistat, the goal is not necessarily to achieve complete PL inhibition, but to strike a balance between reducing lipid absorption and maintaining sufficient enzymatic activity to avoid adverse effects. This balance underscores the therapeutic potential of flavonoids as safer, localized modulators of PL activity.

Some of the most active compounds were also selected to test their type of inhibition against PPL and HPL. The accuracy of linear transformations of the Michaelis–Menten equation for distinguishing between mechanisms of enzymatic inhibition is limited when compared with nonlinear regression, as the transformation can mask the inherent variability of kinetic data [49]. Additionally, modeling the means of repeated experiments instead of using individual data can also lead to the misinterpretation of mechanisms. Therefore, this study used nonlinear regression analysis to determine the kinetic parameters and inhibition types of the most active flavonoids. To achieve this, the Solver supplement of Microsoft Office Excel™ was applied to minimize the sum of squared residuals between the individual raw experimental results and the corresponding values generated by each model.

The kinetic analysis in this study involved sequentially fitting the available models, including those without inhibition, as well as competitive, noncompetitive, uncompetitive, and mixed inhibition. This allowed for the estimation of values for the kinetic constants.

Regarding PPL, based on the results, it was possible to observe that flavone **27** (quercetin), flavone **30**, and flavan-3-ol **41** (EGCG) are competitive inhibitors, while flavone **38** (myricetin) is a mixed-type inhibitor. While the results for flavone **27** (quercetin), flavan-3-ol **41**, (EGCG) and flavone **38** (myricetin) are consistent with previously published findings [46,50,51], it is worth emphasizing that the inhibition type of flavone **30** was investigated in this study for the first time. On the other hand, considering HPL inhibition, it was possible to conclude that flavone **30** is a mixed-type inhibitor and flavone **38** (myricetin) a noncompetitive inhibitor. It was interesting to observe that the mechanisms of enzymatic inhibitions for the same compound varied depending on the enzyme source. These findings can potentially explain the differences in the inhibitory effectiveness of each compound.

Based on the identification of the best kinetic models, the kinetic constants (V_max_, K_m_, K_ic_, and K_iu_) were calculated. To compare the potency of the inhibitors, the K_ic_ values were determined, and for PPL inhibition they followed this order **41** (EGCG, K_ic_ = 21 ± 2) > **27** (quercetin, K_ic_ = 13.1 ± 0.5) > **38** (myricetin, K_ic_ = 12.0 ± 0.8) > **30** (K_ic_ = 1.78 ± 0.04). As expected, considering that the K_ic_ value indicates the compound’s binding affinity to the enzyme, flavonoid **30** exhibited the highest binding affinity to PPL (approximately 10 times higher than the other compounds), followed by **38** (myricetin), **27** (quercetin), and **41** (EGCG). Regarding the potency of the inhibitors against HPL and the determined K_ic_ values, flavonoid **30** (K_ic_ = 1.3 ± 0.3) was approximately 60 times higher than **38** (myricetin, K_ic_ = 74 ± 1). These results may explain the differences in the IC_50_ values, as well as the variations in the inhibition models.

## 5. Conclusions and Future Perspectives

This study aimed to establish a detailed structure–activity relationship for the inhibition of PL using a set of 48 flavonoids, and to investigate whether using PPL or HPL would yield similar inhibition results. The approach included kinetic analysis and the determination of inhibitory mechanisms to understand and explain the binding patterns of the most potent flavonoids. All 48 compounds were docked into the binding site of HPL, allowing for a rigorous analysis of their poses and interaction modes. The docking scores correlated well with the observed inhibitory activities, suggesting that the computational model is effective in predicting the potential of flavonoids to inhibit HPL catalytic activity. This docking study provides valuable insights into the structure–activity relationships of flavonoids as HPL inhibitors, emphasizing the critical role of specific substituents and their positions in determining inhibitory effectiveness. This knowledge can guide the design of more potent HPL inhibitors for therapeutic applications.

The results suggest that the inhibition of PL by flavonoids is highly influenced by the type and position of the substituents. The most effective compounds for both enzymes were the alkylated flavones **28**, **29,** and **30**, along with flavone **38** (myricetin) and flavone **36**. The presence of an extended 3-alkyl group and the C2=C3 double bond of the C-ring, and the pyrogallol group at C-2′, C-3′, and C-4′ of the B-ring, have shown to be favorable for PL catalytic activity inhibition (Figure 16). Flavones **28**, **29**, and **30** from Group D demonstrated the highest docking scores, closely approaching those of orlistat. The presence of alkyl chains at the *ortho* position of the C-ring enhances their inhibitory activity. The docking calculations reveal that flavones **8** (inactive) and **36** (active) are mainly superimposed within the active site, but the additional hydroxyl group on the B-ring allows for potential hydrogen bonding with TYR114 and interactions with PRO180, suggesting the nuanced role of hydroxy groups in enhancing activity. Among the most active flavonoids, flavone **30** presented a competitive inhibition for PPL and mixed-type inhibition for HPL, while flavone **38** (myricetin) presented a mixed-type inhibition for PPL and noncompetitive inhibition for HPL.

The findings of this study highlight that, despite the significant degree of homology between PPL and HPL, it is crucial to include an evaluation with HPL after an initial test with PPL, particularly when working with more complex compounds. This study serves as a preliminary investigation, offering new insights into structural designs that can effectively modulate PL inhibition and highlight the promising potential of flavonoids as therapeutic alternatives. While the initial results suggest that flavonoids exhibit promising inhibitory effects, particularly flavone **30** which demonstrates higher inhibition of HPL, indicating its potential as an effective alternative for obesity management, broader clinical and pharmacological implications, such as bioavailability, metabolic stability, and long-term safety, require further investigation. These findings lay the groundwork for future studies, focusing on the most promising compounds to explore their viability and efficacy in vivo, while addressing their potential limitations and challenges in therapeutic applications. As such, based on these results, additional studies are needed to explore these compounds as potential anti-obesity molecules.

## Figures and Tables

**Figure 4:**
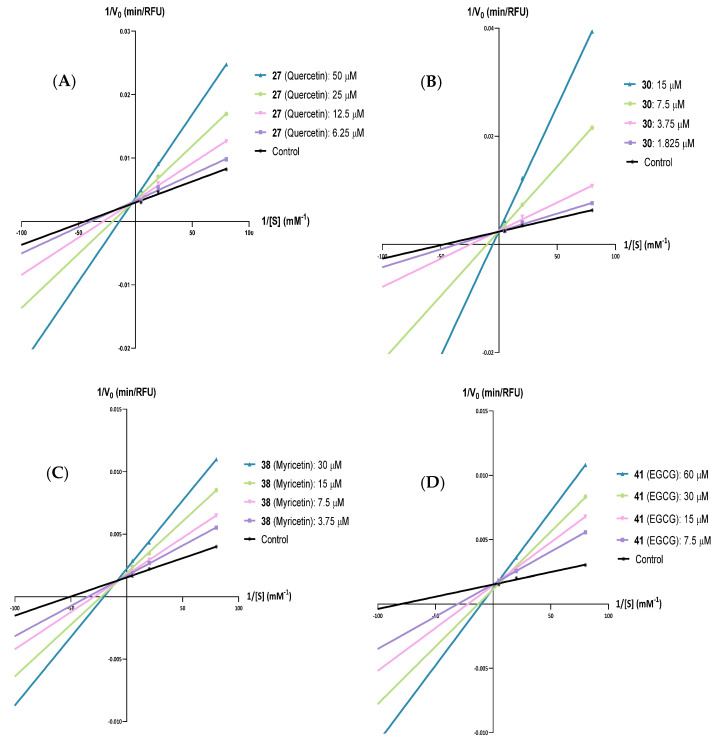
Lineweaver–Burk plots of the 4-methylumbeliferone (4-MU) released in the presence of various concentrations of (**A**) flavone **27** (quercetin), (**B**) flavone **30**, (**C**) flavone **38** (myricetin), and (**D**) flavan-3-ol **41** [(–)-epigallocatechin gallate, EGCG], for porcine pancreatic lipase. Each line was obtained with data from at least three independent experiments.

**Table 1:** Inhibitory catalytic activity of flavonoids against porcine and human pancreatic lipase.

Porcine Pancreatic Lipase						Human Pancreatic Lipase		
4-MUO (μM)	27 (Quercetin) (μM)	30 (μM)	38 (Myricetin) (μM)	41 (EGCG) (μM)		4-MUO (μM)	30 (μM)	38 (Myricetin) (μM)
12.5	0–50.0	0–15.0	0–30.0	0–60.0		12.5	0–10.0	0–50.0
50	0–50.0	0–15.0	0–30.0	0–60.0		50	0–10.0	0–50.0
200	0–50.0	0–15.0	0–30.0	0–60.0		200	0–10.0	0–50.0

(–)-Epigallocatechin gallate (EGCG).

**Table 2:** In vitro inhibitory effect of flavonoids and orlistat on porcine and human PL activity.

	Flavonoids		Ring C	Ring A			Ring B			IC_50_ (μM) ± SEM or PL Inhibition (%) ± SEM	IC_50_ (μM) ± SEM or PL Inhibition (%) ± SEM
			R^3^	R^5^	R^7^	R^8^	R^3^′	R^4^′	R^5^′	Porcine	Human
**GROUP A** *(Flavones)*	**1** *Flavone*	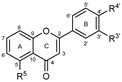	---	---	---	---	---	---	---	<30% ^100 μM^	<30% ^100 μM^
	**2**		---	---	---	---	OH	---	---	<30% ^100 μM^	<30% ^100 μM^
	**3**		---	---	---	---	---	OH	---	<30% ^25 μM^	<30% ^25 μM^
	**4**		---	---	---	---	OH	OH	---	<30% ^100 μM^	<30% ^100 μM^
	**5**		---	OH	---	---	---	---	---	<30% ^50 μM^	<30% ^50 μM^
	**6**		---	OH	---	---	OH	---	---	<30% ^25 μM^	**32.3 ± 0.1%** ^25 μM^
	**7**		---	OH	---	---	---	OH	---	<30% ^25 μM^	<30% ^25 μM^
	**8**		---	OH	---	---	OH	OH	---	**46 ± 2%** ^50 μM^	<30% ^50 μM^
**GROUP B** *(Flavones)*	**9**	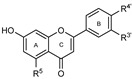	---	---	OH	---	---	---	---	<30% ^50 μM^	<30% ^50 μM^
	**10**		---	---	OH	---	OH	---	---	<30% ^100 μM^	<30% ^100 μM^
	**11**		---	---	OH	---	---	OH	---	(a)	(a)
	**12**		---	---	OH	---	OH	OH	---	(a)	(a)
	**13** *Chrysin*		---	OH	OH	---	---	---	---	<30% ^50 μM^	<30% ^50 μM^
	**14**		---	OH	OH	---	OH	---	---	**73 ± 7**	**54 ± 5**
	**15** *Apigenin*		---	OH	OH	---	---	OH	---	**41 ± 1**	**75 ± 2**
	**16** *Luteolin*		---	OH	OH	---	OH	OH	---	**32 ± 2**	**54 ± 2%** ^50 μM^
	*Isoflavone* **17** *Genistein*	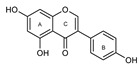	---	OH	OH	---	---	OH	---	<30% ^100 μM^	<30% ^100 μM^
**GROUP C** *(Flavonols)*	**18**	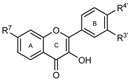	OH	---	---	---	OH	OH	---	**41 ± 4%** ^50 μM^	**44 ± 5%** ^50 μM^
	**19**		OH	---	OH	---	---	---	---	(a)	(a)
	**20**		OH	---	OH	---	OH	---	---	(a)	(a)
	**21**		OH	---	OH	---	---	OH	---	(a)	(a)
	**22** *Fisetin*		OH	---	OH	---	OH	OH	---	(a)	(a)
**GROUP D** *(Flavones)*	**23** *Galangin*	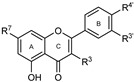	OH	OH	OH	---	---	---	---	**41 ± 1%** ^50 μM^	<30% ^50 μM^
	**24** *Kaempferol*		OH	OH	OH	---	---	OH	---	**79 ± 6**	**39 ± 3%** ^100 μM^
	**25** *Astragalin*		Glu	OH	OH	---	---	OH	---	<30% ^50 μM^	**34 ± 1%** ^50 μM^
	**26** *Populnin*		OH	OH	Glu	---	---	OH	---	<30% ^100 μM^	<30% ^100 μM^
	**27** *Quercetin*		OH	OH	OH	---	OH	OH	---	**27 ± 2**	**46% ± 3**% ^75 μM^
	**28**		C_4_H_9_	OH	OH	---	OH	OH	---	**13 ± 1**	**42 ± 2**
	**29**		C_6_H_13_	OH	OH	---	OH	OH	---	**10.1 ± 0.4**	**16.4 ± 0.7**
	**30**		C_10_H_21_	OH	OH	---	OH	OH	---	**7.3 ± 0.6**	**3.2 ± 0.1**
	**31**		Glu	OH	OH	---	OH	OH	---	<30% ^50 μM^	<30% ^50 μM^
	**32** *Rutin*		Rut	OH	OH	---	OH	OH	---	<30% ^50 μM^	**34 ± 1%** ^50 μM^
	**33** *Quercimeritrin*		OH	OH	Glu	---	OH	OH	---	<30% ^50 μM^	<30% ^50 μM^
	**34** *Spiraeoside*		OH	OH	OH	---	OH	Glu	---	<30% ^50 μM^	<30% ^50 μM^
	**35**		Glu	OH	OH	---	OH	Glu	---	<30% ^50 μM^	<30% ^50 μM^
**GROUP E** *(Flavones)*	**36**	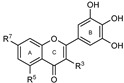	---	OH	---	---	OH	OH	OH	**20 ± 3**	**7 ± 1**
	**37** *Robinetin*		OH	---	OH	---	OH	OH	OH	(a)	(a)
	**38**		OH	OH	OH	---	OH	OH	OH	**16 ± 1**	**25 ± 2**
**GROUP F** *(Flavan-3-ols)*	**39** *(–)-Epicatechin*	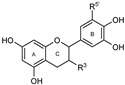	OH	OH	OH	---	OH	OH	---	<30% 100 μM	<30% 100 μM
	**40** *(–)-Epigallocatechin*		OH	OH	OH	---	OH	OH	OH	<30% 100 μM	<30% 100 μM
	**41** *(–)-Epigallocatechin gallate*		Gal	OH	OH	---	OH	OH	OH	32 ± 4	<30% 100 μM
**GROUP G** *(Flavones)*	**42** *Norwogonin*	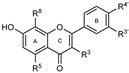	---	OH	OH	OH	---	---	---	**98 ± 9**	**79 ± 3**
	**43**		---	---	OH	OH	OH	OH	---	<30% ^50 μM^	<30% ^50 μM^
	**44**		OH	---	OH	OH	---	OH	---	**42 ± 2%** ^50 μM^	<30% ^50 μM^
	**45** *Melanoxetin*		OH	---	OH	OH	OH	OH	---	<30% ^25 μM^	<30% ^25 μM^
	**46** *Herbacetin*		OH	OH	OH	OH	---	OH	---	**46 ± 4%** ^50 μM^	**39 ± 3%** ^50 μM^
	**47** *Gossypetin*		OH	OH	OH	OH	OH	OH	---	**36 ± 3%** ^50 μM^	**37 ± 1**% ^50 μM^
	**48** *Gossypin*		OH	OH	OH	Glu	OH	OH	---	<30% ^12.5 μM^	<30% ^12.5 μM^
	**Orlistat**		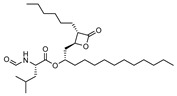							**0.0043 ± 0.0003**	**0.00068 ± 0.00003**
Glucosyl (Glu): 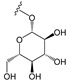			Rutinosyl (Rut): 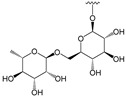							Galloyl (Gal): 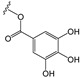	

(a) The effect of this flavonoid on pancreatic lipase catalytic activity was not evaluated, due to its intrinsic fluorescence. Pancreatic lipase (PL).

**Table 3:** Inhibition model and V_max_, K_m_, K_ic_, and K_iu_ ± SEM, for porcine pancreatic lipase inhibition by the selected flavonoids.

Compound	Inhibition Model	V_max_	K_m_	K_ic_	K_iu_
**27** (Quercetin)	Competitive	354 ± 3	26.0 ± 0.8	13.1 ± 0.5	-
**30**	Competitive	507 ± 4	26.0 ± 0.7	1.78 ± 0.04	-
**38** (Myricetin)	Mixed	655 ± 5	23.0 ± 0.7	12.0 ± 0.8	58 ± 3
**41** (EGCG)	Competitive	680 ± 7	21 ± 2	21 ± 3	-

(–)-Epigallocatechin gallate (EGCG).

**Table 4:** Inhibition model and V_max_, K_m_, K_ic_, and K_iu_ ± SEM, for human pancreatic lipase inhibition by the selected flavonoids.

Compound	Inhibition Model	V_max_	K_m_	K_ic_	K_ic_
**30**	Mixed	260 ± 10	14 ± 2	1.3 ± 0.3	16 ± 3
**38** (Myricetin)	Noncompetitive	230 ± 1	9.6 ± 0.1	74 ± 1	74 ± 1

**Table 5:** Molecular docking scores for the interaction of active flavonoids with human pancreatic lipase obtained with GNINA (CNN affinity), from lowest to highest.

Compound	15	36	14	38	42	28	29	30	Orlistat
GNINA Score (CNN affinity)	5.493	5.639	5.728	6.167	6.328	6.796	7.015	7.455	7.693

## Data Availability

The original contributions presented in this study are included in the article/Supplementary Material. Further inquiries can be directed to the corresponding author(s).

## Supplementary Materials

The following supporting information can be downloaded at: https://www.mdpi.com/article/10.3390/pharmaceutics17020163/s1, Figure S1: Mean values of the slopes (y values) and respective standard deviations as results of the in vitro inhibition of porcine pancreatic lipase (0.04 mg/mL) by flavonoid 27 (quercetin) (0–50 μM) using three substrate concentrations (x values: 12.5, 50 and 200 μM); Figure S2: Sum of the squares (sum) of the different models (without inhibition, competitive inhibition, noncompetitive inhibition, uncompetitive inhibition, and mixed inhibition) determined from the results obtained from porcine pancreatic lipase inhibition by flavonoid 27 (quercetin); Figure S3: Comparison of the different models (without inhibition, competitive inhibition, noncompetitive inhibition, uncompetitive inhibition, and mixed inhibition), based on the porcine pancreatic lipase inhibition by flavonoid 27 (quercetin); Figure S4: Error parameters determination (V_max_, K_m_ and K_ic_) for competitive inhibition model of porcine pancreatic lipase by flavonoid 27 (quercetin), through “Jackknife” procedure; Figure S5: Mean values of the slopes (y values) and respective standard deviations as results of the in vitro inhibition of porcine pancreatic lipase (0.04 mg/mL) by flavonoid 30 (0–15 μM) using three substrate concentrations (x values: 12.5, 50 and 200 μM); Figure S6: Sum of the squares (sum) of the different models (without inhibition, competitive inhibition, noncompetitive inhibition, uncompetitive inhibition, and mixed inhibition) determined from the results obtained from porcine pancreatic lipase inhibition by flavonoid 30; Figure S7: Comparison of the different models (without inhibition, competitive inhibition, noncompetitive inhibition, uncompetitive inhibition, and mixed inhibition), based on the porcine pancreatic lipase inhibition by flavonoid 30; Figure S8: Error parameters determination (V_ma_x, K_m_ and K_ic_) for competitive inhibition model of porcine pancreatic lipase by flavonoid 30, through “Jackknife” procedure; Figure S9: Mean values of the slopes (y values) and respective standard deviations as results of the in vitro inhibition of porcine pancreatic lipase (0.04 mg/mL) by flavonoid 38 (0–30 μM) using three substrate concentrations (x values: 12.5, 50 and 200 μM); Figure S10: Sum of the squares (sum) of the different models (without inhibition, competitive inhibition, noncompetitive inhibition, uncompetitive inhibition, and mixed inhibition) determined from the results obtained from porcine pancreatic lipase inhibition by flavonoid 38; Figure S11: Comparison of the different models (without inhibition, competitive inhibition, noncompetitive inhibition, uncompetitive inhibition, and mixed inhibition), based on the porcine pancreatic lipase inhibition by flavonoid 38; Figure S12: Error parameters determination (V_max_, Km, K_ic_ and K_iu_) for mixed inhibition model of porcine pancreatic lipase by flavonoid 38, through “Jackknife” procedure; Figure S13: Mean values of the slopes (y values) and respective standard deviations as results of the in vitro inhibition of porcine pancreatic lipase (0.04 mg/mL) by flavonoid 41 (EGCG) (0–60 μM) using three substrate concentrations (x values: 12.5, 50 and 200 μM); Figure S14: Sum of the squares (sum) of the different models (without inhibition, competitive inhibition, noncompetitive inhibition, uncompetitive inhibition, and mixed inhibition) determined from the results obtained from porcine pancreatic lipase inhibition by flavonoid 41 (EGCG); Figure S15: Comparison of the different models (without inhibition, competitive inhibition, noncompetitive inhibition, uncompetitive inhibition, and mixed inhibition), based on the porcine pancreatic lipase inhibition by flavonoid 41 (EGCG); Figure S16: Error parameters determination (V_max_, K_m_ and K_ic_) for competitive inhibitions model of porcine pancreatic lipase by flavonoid 41 (EGCG), through “Jackknife” procedure; Figure S17: Mean values of the slopes (y values) and respective standard deviations as results of the in vitro inhibition of human pancreatic lipase (2 U/mL) by flavonoid 30 (0–10 μM) using three substrate concentrations (x values: 12.5, 50 and 200 μM); Figure S18: Sum of the squares (sum) of the different models (without inhibition, competitive inhibition, noncompetitive inhibition, uncompetitive inhibition, and mixed inhibition) determined from the results obtained from human pancreatic lipase inhibition by flavonoid 30; Figure S19: Comparison of the different models (without inhibition, competitive inhibition, noncompetitive inhibition, uncompetitive inhibition, and mixed inhibition), based on the human pancreatic lipase inhibition by flavonoid 30; Figure S20. Error parameters determination (V_max_, K_m_ and K_ic_) for mixed inhibition model of human pancreatic lipase by flavonoid 30, through “Jackknife” procedure; Figure S21: Mean values of the slopes (y values) and respective standard deviations as results of the in vitro inhibition of human pancreatic lipase (2 U/mL) by flavonoid 38 (0–50 μM) using three substrate concentrations (x values: 12.5, 50 and 200 μM); Figure S22: Sum of the squares (sum) of the different models (without inhibition, competitive inhibition, noncompetitive inhibition, uncompetitive inhibition, and mixed inhibition) determined from the results obtained from human pancreatic lipase inhibition by flavonoid 38; Figure S23: Comparison of the different models (without inhibition, competitive inhibition, noncompetitive inhibition, uncompetitive inhibition, and mixed inhibition), based on the human pancreatic lipase inhibition by flavonoid 38; Figure S24: Error parameters determination (V_max_, K_m_, K_ic_ and K_iu_) for noncompetitive inhibition model of human pancreatic lipase by flavonoid 38, through “Jackknife” procedure; Tabel S1: Molecular docking scores for the interaction of all tested flavonoids with human pancreatic lipase obtained with GNINA (CNN Affinity), from lowest to highest.

## Abbreviations

4-MU	4-methylumbeliferone
4-MUO	4-methylumbelliferyl oleate
AUC	Area under the curve
BMI	Body mass index
DMSO	Dimethyl sulfoxide
EGCG	(–)-epigallocatechin gallate
HPL	Human pancreatic lipase
PL	Pancreatic lipase
PPL	Porcine pancreatic lipase
RFU	Relative fluorescence unit
RMSD	Root mean square deviation
ROC	Receiver operating characteristic
Tris	Tris(hydroxymethyl)aminomethane
WHO	World Health Organization
WOF	World Obesity Federation

## References

[1] Jensen M.D., Ryan D.H., Apovian C.M., Ard J.D., Comuzzie A.G., Donato K.A., Hu F.B., Hubbard V.S., Jakicic J.M., Kushner R.F. (2014). 2013 AHA/ACC/TOS guideline for the management of overweight and obesity in adults: A report of the American College of Cardiology/American Heart Association Task Force on Practice Guidelines and The Obesity Society. J. Am. Coll. Cardiol..

[2] Bray G.A., Kim K.K., Wilding J.P.H., World Obesity Federation (2017). Obesity: A chronic relapsing progressive disease process. A position statement of the World Obesity Federation. Obes. Rev..

[3] Blüher M. (2019). Obesity: Global epidemiology and pathogenesis. Nat. Rev. Endocrinol..

[4] Collaboration P.S. (2009). Body-mass index and cause-specific mortality in 900 000 adults: Collaborative analyses of 57 prospective studies. Lancet.

[5] Apovian C.M. (2016). Obesity: Definition, comorbidities, causes, and burden. AJMC.

[6] World Obesity Federation (2022). World Obesity Atlas 2022.

[7] World Obesity Federation (2023). World Obesity Atlas 2023.

[8] Engin A. (2017). The definition and prevalence of obesity and metabolic syndrome. Obesity and Lipotoxicity.

[9] World Health Organization (2019). Global Action Plan on Physical Activity 2018–2030: More Active People for a Healthier World.

[10] Demirci N., Demirci N., Yildirim İ., Demirci P.T., Ersroz Y. (2018). Why should we do physical activity? More active people for a healthier world. Int. J. Disabil. Sports Heal. Sci..

[11] (1998). Clinical Guidelines on the Identification, Evaluation, and Treatment of Overweight and Obesity in Adults: The Evidence Report.

[12] Wadden T.A., Bray G.A. (2018). Handbook of Obesity Treatment.

[13] Thomson A.B.R., De Pover A., Keelan M., Jarocka-Cyrta E., Clandinin M.T. (1997). Inhibition of lipid absorption as an approach to the treatment of obesity. Meth. Enzymol..

[14] Lowe M.E. (1997). Structure and function of pancreatic lipase and colipase. Annu. Rev. Nutr..

[15] Lowe M.E., Rosenblum J.L., Strauss A.W. (1989). Cloning and characterization of human pancreatic lipase cDNA. J. Biol. Chem..

[16] Chapus C., Rovery M., Sarda L., Verger R. (1988). Minireview on pancreatic lipase and colipase. Biochimie.

[17] Gargouri Y., Bensalah A., Douchet I., Verger R. (1995). Kinetic behaviour of pancreatic lipase in five species using emulsions and monomolecular films of synthetic glycerides. Biochim. Biophys. Acta..

[18] Abousalham A., Verger R. (2000). Egg yolk lipoproteins as substrates for lipases. Biochim. Biophys. Acta Mol. Cell. Biol. Lipids.

[19] Chanoine J.-P., Hampl S., Jensen C., Boldrin M., Hauptman J. (2005). Effect of orlistat on weight and body composition in obese adolescents: A randomized controlled trial. JAMA.

[20] Tan H.C., Dampil O.A., Marquez M.M. (2022). Efficacy and safety of semaglutide for weight loss in obesity without diabetes: A systematic review and meta-analysis. J. ASEAN Fed. Endocr. Soc..

[21] Qi X. (2018). Review of the clinical effect of orlistat. IOP Conf. Ser. Mater. Sci. Eng..

[22] Yao L.H., Jiang Y.M., Tomás-Barberán F.A., Datta N., Singanusong R., Chen S.S. (2004). Flavonoids in food and their health benefits. Plant Foods Hum. Nutr..

[23] Li A.-N., Li S., Zhang Y.-J., Xu X.-R., Chen Y.-M., Li H.-B. (2014). Resources and biological activities of natural polyphenols. Nutrients.

[24] Hasnat H., Shompa S.A., Islam M.M., Alam S., Richi F.T., Emon N.U., Ashrafi S., Ahmed N.U., Chowdhury N.R., Fatema N. (2024). Flavonoids: A treasure house of prospective pharmacological potentials. Heliyon.

[25] Claudine M., Scalbert A., Morand C., Rémésy C., Jiménez L. (2024). Polyphenols: Food sources and bioavailability. Am. J. Clin. Nutr..

[26] Tang Z., Zhang Q. (2022). The potential toxic side effects of flavonoids. Biocell.

[27] Rocha S., Rufino A.T., Freitas M., Silva A.M.S., Carvalho F., Fernandes E. (2023). Methodologies for assessing pancreatic lipase catalytic activity: A review. Crit. Rev. Anal. Chem..

[28] Ribeiro D., Freitas M., Tomé S.M., Silva A.M.S., Porto G., Fernandes E. (2013). Modulation of human neutrophils’ oxidative burst by flavonoids. Eur. J. Med. Chem..

[29] Seixas R.S.G.R., Pinto D.C.G.A., Silva A.M.S., Cavaleiro J.A.S. (2008). Synthesis of Novel 3-Alkyl-3′, 4′, 5, 7-Tetrahydroxyflavones. Aust. J. Chem..

[30] Sousa J.L.C., Proenca C., Freitas M., Fernandes E., Silva A.M.S. (2016). New polyhydroxylated flavon-3-ols and 3-hydroxy-2-styrylchromones: Synthesis and ROS/RNS scavenging activities. Eur. J. Med. Chem..

[31] Zhang B., Deng Z., Ramdath D., Tang Y., Chen P.X., Liu R., Liu Q., Tsao R. (2015). Phenolic profiles of 20 Canadian lentil cultivars and their contribution to antioxidant activity and inhibitory effects on α-glucosidase and pancreatic lipase. Food Chem..

[32] Ivanov S.A., Nomura K., Malfanov I.L., Sklyar I.V., Ptitsyn L.R. (2011). Isolation of a novel catechin from Bergenia rhizomes that has pronounced lipase-inhibiting and antioxidative properties. Fitoterapia.

[33] Freitas M., Proença C., Ribeiro D., Quinaz-Garcia M.B., Araújo A.N., Fernandes E. (2023). Assessment of α-amylase activity in a microanalysis system: Experimental optimization and evaluation of type of inhibition. J. Chem. Educ..

[34] Marchand L.R., Pirard B., Ertl P., Sirockin F. (2021). CAVIAR: A method for automatic cavity detection, description and decomposition into subcavities. J. Comput. Aided Mol. Des..

[35] Eberhardt J., Santos-Martins D., Tillack A.F., Forli S. (2021). AutoDock Vina 1.2.0: New docking methods, expanded force field, and python bindings. J. Chem. Inf. Model..

[36] Friesner R.A., Murphy R.B., Repasky M.P., Frye L.L., Greenwood J.R., Halgren T.A., Sanschagrin P.C., Mainz D.T. (2006). Extra precision glide: Docking and scoring incorporating a model of hydrophobic enclosure for protein– ligand complexes. J. Med. Chem..

[37] McNutt A.T., Francoeur P., Aggarwal R., Masuda T., Meli R., Ragoza M., Sunseri J., Koes D.R. (2021). GNINA 1.0: Molecular docking with deep learning. J. Cheminf..

[38] Salentin S., Schreiber S., Haupt V.J., Adasme M.F., Schroeder M. (2015). PLIP: Fully automated protein–ligand interaction profiler. Nucleic Acids Res..

[39] Raschka S. (2017). BioPandas: Working with molecular structures in pandas DataFrames. J. Open Source Softw..

[40] Heck A.M., Yanovski J.A., Calis K.A. (2000). Orlistat, a new lipase inhibitor for the management of obesity. Pharmacotherapy.

[41] Bauer E., Jakob S., Mosenthin R. (2005). Principles of physiology of lipid digestion. Asian-Australas. J. Anim. Sci..

[42] Zdrazil B., Felix E., Hunter F., Manners E.J., Blackshaw J., Corbett S., Veij M., Ioannidis H., Lopez D.M., Mosquera J.F. (2024). The ChEMBL Database in 2023: A drug discovery platform spanning multiple bioactivity data types and time periods. Nucleic Acids Res..

[43] Bray G.A. (2014). Medical treatment of obesity: The past, the present and the future. Best Pract. Res. Clin. Obstet. Gynaecol..

[44] Yanovski S.Z., Yanovski J.A. (2014). Long-term drug treatment for obesity: A systematic and clinical review. JAMA.

[45] Park J.-Y., Kim C.S., Park K.-M., Chang P.-S. (2019). Inhibitory characteristics of flavonol-3-O-glycosides from *Polygonum aviculare* L. (common knotgrass) against porcine pancreatic lipase. Sci. Rep..

[46] Nakai M., Fukui Y., Asami S., Toyoda-Ono Y., Iwashita T., Shibata H., Mitsunaga T., Hashimoto F., Kiso Y. (2005). Inhibitory effects of oolong tea polyphenols on pancreatic lipase in vitro. J. Agric. Food Chem..

[47] Yuda N., Tanaka M., Suzuki M., Asano Y., Ochi H., Iwatsuki K. (2012). Polyphenols extracted from black tea (Camellia sinensis) residue by hot-compressed water and their inhibitory effect on pancreatic lipase in vitro. J. Food Sci..

[48] Cha K.H., Song D.-G., Kim S.M., Pan C.-H. (2012). Inhibition of gastrointestinal lipolysis by green tea, coffee, and gomchui (Ligularia fischeri) tea polyphenols during simulated digestion. J. Agric. Food Chem..

[49] Bezerra R.M.F., Fraga I., Dias A.A. (2013). Utilization of integrated Michaelis–Menten equations for enzyme inhibition diagnosis and determination of kinetic constants using Solver supplement of Microsoft Office Excel. Comput. Methods Programs Biomed..

[50] Martinez-Gonzalez A.I., Alvarez-Parrilla E., Díaz-Sánchez Á.G., de la Rosa L.A., Núñez-Gastélum J.A., Vazquez-Flores A.A., Gonzalez-Aguilar G.A. (2017). In vitro inhibition of pancreatic lipase by polyphenols: A kinetic, fluorescence spectroscopy and molecular docking study. Food Technol. Biotechnol..

[51] Rahim A.T.M.A., Takahashi Y., Yamaki K. (2015). Mode of pancreatic lipase inhibition activity in vitro by some flavonoids and non-flavonoid polyphenols. Food Res. Int..

