# Supplementary material for: Flavonoids as Potential Modulators of Pancreatic Lipase Catalytic Activity
Source: Pharmaceutics. 2025 Jan 25;17(2):163. doi: 10.3390/pharmaceutics17020163 (PMC11859905; doi:10.3390/pharmaceutics17020163)
Supplement: Supplementary file 1 [file pharmaceutics-17-00163-s001.zip › pharmaceutics-3350657-supplementary.pdf]

Supplementary Materials: Flavonoids as Potential Modulators of Pancreatic Lipase Catalytic Activity

Sílvia Rocha, Carina Proença, Alberto N. Araújo, Marisa Freitas, Ismael Rufino, Natália Aniceto, Artur M. S. Silva, Félix Carvalho, Rita C. Guedes and Eduarda Fernandes

PORCINE PANCREATIC LIPASE
Flavonoid 27 (Quercetin)

Nonlinear regression using Solver

values of x	values of y	concentration of inhibitor (µM)	slopes (replicates)			standard deviation y_{exp}
12,5	121,67	0	113,29	188,39	63,34	62,95
50	214,45	0	170,52	281,11	191,71	58,70
200	332,33	0	296,28	423,13	277,57	79,19
12,5	102,23	6,25	93,93	162,78	49,99	56,85
50	185,61	6,25	136,90	247,23	172,70	56,28
200	304,37	6,25	239,07	387,93	286,11	76,09
12,5	79,21	12,5	72,01	125,32	40,28	42,97
50	166,44	12,5	131,58	216,46	151,28	44,42
200	276,34	12,5	235,14	345,68	248,18	60,41
12,5	59,00	25	53,88	98,33	24,80	37,03
50	141,45	25	112,72	186,75	124,87	39,70
200	248,89	25	203,85	308,40	234,41	53,76
12,5	40,40	50	29,77	71,44	19,99	27,32
50	110,06	50	74,74	165,03	90,41	48,25
200	204,25	50	178,60	268,81	165,34	56,30

Figure S1. Mean values of the slopes (y values) and respective standard deviations as results of the in vitro inhibition of porcine pancreatic lipase (0.04 mg/mL) by flavonoid **27** (quercetin) (0–50 µM) using three substrate concentrations (x values: 12.5, 50 and 200 µM).

Without Inhibition	Competitive Inhibition	Noncompetitive Inhibition	Uncompetitive Inhibition	Mixed Inhibition
$v_{inic} = \frac{V_{max} \times \lvert S\rvert}{K_m + \lvert S\rvert}$	$v_{inic} = \frac{V_{max} \times \lvert S\rvert}{K_m \times (1 + \frac{\lvert I\rvert}{K_{ic}}) + \lvert S\rvert}$	$v_{inic} = \frac{V_{max} \times \lvert S\rvert}{K_m \times (1 + \frac{\lvert I\rvert}{K_{ic}}) + \lvert S\rvert \times (1 + \frac{\lvert I\rvert}{K_{iu}})}$	$v_{inic} = \frac{V_{max}(S)}{K_m + (S) \times (1 + \frac{\lvert I\rvert}{K_{iu}})}$	$v_{inic} = \frac{V_{max} \times \lvert S\rvert}{K_m \times (1 + \frac{\lvert I\rvert}{K_{ic}}) + \lvert S\rvert \times (1 + \frac{\lvert I\rvert}{K_{iu}})}$

$(y_{exp} - y_{cal})^2$	Ycalc	$(y_{exp} - y_{cal})^2$	Ycalc	$(y_{exp} - y_{cal})^2$	Ycalc	$(y_{exp} - y_{cal})^2$	Ycalc	$(y_{exp} - y_{cal})^2$	Ycalc
15879,7123	70,1774	8114,6042	113,7124	11042,9487	89,4314	13327,7241	79,2333	8671,3063	105,8949
12250,2273	172,1782	7785,3455	231,7194	6895,4113	215,7501	7243,0288	203,6038	7499,0702	228,6918
24028,6042	270,4516	13675,2031	312,8984	12547,0194	333,5221	12565,8864	335,1053	12859,1881	322,0570
9545,7788	70,1774	7276,4981	85,7695	7756,0008	81,4747	8321,9355	77,3417	7253,5426	86,0036
6876,8652	172,1782	6852,2969	198,7351	6694,8710	196,5548	6441,8911	191,5643	6971,0422	200,1651
15029,5897	270,4516	11774,0214	296,2972	11579,4074	303,8486	11579,9715	303,6914	11647,3282	299,5817
3937,9951	70,1774	4015,1432	68,8506	3751,2299	74,8182	3733,8287	75,5384	3832,2875	72,4033
4045,7712	172,1782	4117,1256	173,9710	4539,6531	180,4961	4571,5347	180,8692	4345,4936	177,9660
7402,3667	270,4516	7374,5181	281,3687	7320,1979	279,0239	7303,7971	277,6625	7339,6586	280,0387
3117,3667	70,1774	3020,8320	49,3723	2827,2000	64,3098	3263,1032	72,1726	2790,5490	55,0064
5984,7760	172,1782	3165,9175	139,2640	3714,5010	155,1451	4506,8674	162,7017	3204,8014	145,6578
7174,7871	270,4516	5915,2128	255,6116	6025,2571	239,8344	6201,0621	237,0312	5783,5725	247,7192
4152,8021	70,1774	1729,2627	31,5314	1781,5317	50,2066	3500,1476	66,2674	1524,8594	37,1525
16231,2696	172,1782	4987,0593	99,5456	5022,4329	121,1216	6594,5073	135,4843	4686,1234	106,8591
19488,1990	270,4516	6757,9915	216,0552	7207,9071	187,2384	7648,1107	183,3661	6366,5744	201,2632
155146,1109	sum	96561,0319	sum	98705,5692	sum	106803,3963	sum	94775,3975	sum

Without Inhibition		Competitive Inhibition		Noncompetitive Inhibition		Uncompetitive Inhibition		Mixed Inhibition	
V_{max} =	333,9962	V_{max} =	354,2692	V_{max} =	407,7074	V_{max} =	427,0433	V_{max} =	372,7883
K_m =	46,9914	K_m =	26,4436	K_m =	44,4860	K_m =	54,8711	K_m =	31,5045
		K_{ic} =	13,0264	K_{iu}= K_{ic}=	63,9984	K_{iu} =	47,4134	K_{ic} =	20,3031
								K_{iu} =	162,9772

Figure S2. Sum of the squares (sum) of the different models (without inhibition, competitive inhibition, noncompetitive inhibition, uncompetitive inhibition, and mixed inhibition) determined from the results obtained from porcine pancreatic lipase inhibition by flavonoid **27** (quercetin).

Models comparison based on F test

	$(y_{exp} - y_{cal})^2$	p	N	N-p	$f_{0,05}$	Fcalc	ΔSS	Δdf	ΔSS / Δdf
Without Inhibition	155146,1109	2	45	43					
Competitive Inhibition	96561,0319	3	45	42	1,667	25,48	0,019	0,024	0,772
Noncompetitive Inhibition	98705,5692	3	45	42	1,671	24,02	0,041	0,024	1,700
Uncompetitive Inhibition	106803,3963	3	45	42	1,671	19,01	0,127	0,024	5,203
Mixed Inhibition	94775,3975	4	45	41	1,678	13,06			
	Criterium 1					Criterium 2			Criterium 3
	Lower ΔSS for the same N-p					Higher Fcalc			Evaluate if mixed inhibition is better than other inhibition

If Δ SS/Δ df value is higher than $f_{0,05}$ value, mixed inhibition is prefered

Comparison based on Akaike:

	AIC c	ΔAICc			
Without Inhibition	373,1038				
Competitive Inhibition	354,1594	-18,94			
Noncompetitive Inhibition	355,1479	-17,96			
Uncompetitive Inhibition	358,6961	-14,41			
Mixed Inhibition	355,8305	-17,27	1,67	0,68	-2,87

Figure S3. Comparison of the different models (without inhibition, competitive inhibition, noncompetitive inhibition, uncompetitive inhibition, and mixed inhibition), based on the porcine pancreatic lipase inhibition by flavonoid **27** (quercetin).

Uncertainties calculation by the "Jackknife" procedure (95% level of confidance)

Eliminated number	V_{max}	K_m	K_{ic}
1	358,51	28,86	14,35
2	354,72	23,64	10,84
3	336,23	24,14	13,78
4	358,98	28,34	13,57
5	352,97	25,28	12,58
6	348,96	25,65	13,03
7	356,54	27,13	13,03
8	353,10	25,97	13,08
9	356,76	26,88	13,15
10	355,76	26,72	12,84
11	354,74	26,52	12,92
12	356,37	26,96	13,42
13	355,13	26,52	12,82
14	356,60	26,56	12,29
15	355,74	27,37	14,42

V_{max} error:	3,04
K_m error:	0,76
K_{ic} error:	0,48

Figure S4. Error parameters determination (V_{max}, K_m and K_{ic}) for competitive inhibition model of porcine pancreatic lipase by flavonoid **27** (quercetin), through "Jackknife" procedure.

Flavonoid 30

Nonlinear regression using Solver

values of x	values of y	concentration of inhibitor (µM)	slopes (replicates)				standard deviation y_{exp}
12,5	158,21	0	128,363	211,425	134,837		46,20
50	319,07	0	254,548	286,616	416,034		85,49
200	467,45	0	442,941	372,426	586,993		109,36
12,5	131,13	1,825	123,861	145,241	100,541	154,883	24,17
50	248,04	1,825	226,710	222,332	265,826	277,301	27,62
200	402,06	1,825	374,303	301,144	457,643	475,137	80,38
12,5	92,75	3,75	87,164	94,037	74,809	114,978	16,82
50	196,40	3,75	193,720	182,775	212,542	196,578	12,29
200	369,31	3,75	344,084	368,069	395,779		25,87
12,5	46,34	7,5	50,366	52,342	34,028	48,606	8,35
50	136,31	7,5	136,476	121,174	137,442	150,136	11,85
200	280,49	7,5	266,928	210,241	315,205	329,579	53,96
12,5	27,07	15	30,388	22,774	28,049		3,90
50	81,52	15	84,950	83,420	76,895	80,812	3,52
200	230,10	15	222,527	233,137	234,64		6,60

Figure S5. Mean values of the slopes (y values) and respective standard deviations as results of the in vitro inhibition of porcine pancreatic lipase (0.04 mg/mL) by flavonoid **30** (0–15 µM) using three substrate concentrations (x values: 12.5, 50 and 200 µM).

Without Inhibition	Competitive Inhibition	Noncompetitive Inhibition	Uncompetitive Inhibition	Mixed Inhibition
$v_{inic} = \frac{V_{max} \times \lvert S \rvert}{K_m + \lvert S \rvert}$	$v_{inic} = \frac{V_{max} \times \lvert S \rvert}{K_m \times (1 + \frac{\lvert I \rvert}{K_{ic}}) + \lvert S \rvert}$	$v_{inic} = \frac{V_{max} \times \lvert S \rvert}{K_m \times (1 + \frac{\lvert I \rvert}{K_{ic}}) + \lvert S \rvert \times (1 + \frac{\lvert I \rvert}{K_{iu}})}$	$v_{inic} = \frac{V_{max}(S)}{K_m + (S) \times (1 + \frac{\lvert I \rvert}{K_{iu}})}$	$v_{inic} = \frac{V_{max} \times \lvert S \rvert}{K_m \times (1 + \frac{\lvert I \rvert}{K_{ic}}) + \lvert S \rvert \times (1 + \frac{\lvert I \rvert}{K_{iu}})}$

$(y_{exp} - y_{cal})^2$	Ycalc	$(y_{exp} - y_{cal})^2$	Ycalc	$(y_{exp} - y_{cal})^2$	Ycalc	$(y_{exp} - y_{cal})^2$	Ycalc	$(y_{exp} - y_{cal})^2$	Ycalc
25148,8727	74,7819	4441,5420	165,7926	8697,4441	119,7876	14188,0610	100,7074	4268,9771	158,2189
56828,2744	200,4491	15368,7810	334,8817	15481,7841	302,1012	20175,1295	276,0282	15176,6926	332,7082
68415,2593	345,6687	24889,0347	449,4881	25144,2785	487,6482	25279,9838	488,7387	24117,4458	459,3567
14452,9482	74,7819	6071,4149	98,2698	5602,8843	100,1032	6555,0396	96,4790	5201,3202	101,7654
11348,8496	200,4491	2289,7433	248,6178	2366,3991	252,4576	2298,8647	246,4262	2399,5681	253,3136
32102,6492	345,6687	19385,4414	402,6121	19503,3392	407,5141	19387,9088	403,0186	19393,2128	403,5572
2139,7986	74,7819	3154,1881	68,7399	1069,7445	85,3153	849,3405	92,3874	2263,9047	73,9382
518,8783	200,4491	456,6941	195,4989	1861,0540	215,1630	2949,3735	221,3835	596,0221	202,3746
3015,3246	345,6687	1469,0878	362,7130	2790,1138	347,3136	3898,3088	340,0999	1741,3616	357,7224
3445,7495	74,7819	244,4040	43,3584	1795,2983	66,2500	6293,4815	85,3372	223,4764	48,2410
16878,4020	200,4491	433,6230	138,0432	4209,6609	167,0808	9827,5529	184,7992	753,1462	145,4117
25727,6837	345,6687	10948,8356	304,0208	9199,2728	269,6998	10286,2185	260,7873	9351,3207	292,9141
6859,6116	74,7819	44,0322	24,9405	1081,2894	45,7863	6648,1517	74,0374	36,2091	28,4591
56614,5042	200,4491	154,8357	86,9407	4648,4153	115,4720	13204,6958	138,8940	567,8974	93,0370
40154,6453	345,6687	87,6994	229,6874	5818,3064	186,3935	8280,6261	177,8410	770,5805	215,0083
363651,4512	sum	89439,3572	sum	109269,2848	sum	150122,7367	sum	86861,1355	sum

Without Inhibition		Competitive Inhibition		Noncompetitive Inhibition		Uncompetitive Inhibition		Mixed Inhibition	
V_{max} =	455,7210	V_{max} =	507,3666	V_{max} =	613,1853	V_{max} =	657,6764	V_{max} =	526,1134
K_m =	63,6750	K_m =	25,7531	K_m =	51,4867	K_m =	69,1321	K_m =	29,0653
		K_{ic} =	1,7881	$K_{iu} = K_{ic}$ =	9,2809	K_{iu} =	6,3763	K_{ic} =	2,3592
								K_{iu} =	39,7214

Figure S6. Sum of the squares (sum) of the different models (without inhibition, competitive inhibition, noncompetitive inhibition, uncompetitive inhibition, and mixed inhibition) determined from the results obtained from porcine pancreatic lipase inhibition by flavonoid **30**.

Models comparison based on F test

	$(y_{exp} - y_{cal})^2$	p	N	N-p	$f_{0,05}$	Fcalc	ΔSS	Δdf	ΔSS / Δdf
Without Inhibition	363651,4512	2	54	52					
Competitive Inhibition	89439,3572	3	54	51	1,589	156,36	0,030	0,020	1,484
Noncompetitive Inhibition	109269,2848	3	54	51	1,592	118,73	0,258	0,020	12,899
Uncompetitive Inhibition	150122,7367	3	54	51	1,592	72,54	0,728	0,020	36,415
Mixed Inhibition	86861,1355	4	54	50	1,597	79,66			
	Criterium 1					Criterium 2			Criterium 3
	Lower ΔSS for the same N-p					Higher Fcalc			Evaluate if mixed inhibition is better than other inhibition

If Δ SS/Δ df value is higher than $f_{0,05}$ value, mixed inhibition is prefered

Comparison based on Akaike:

	AIC c	ΔAICc			
Without Inhibition	482,4698				
Competitive Inhibition	409,0502	-73,42			
Noncompetitive Inhibition	419,8640	-62,61			
Uncompetitive Inhibition	437,0164	-45,45			
Mixed Inhibition	409,8864	-72,58	0,84	-9,98	-27,13

Figure S7. Comparison of the different models (without inhibition, competitive inhibition, noncompetitive inhibition, uncompetitive inhibition, and mixed inhibition), based on the porcine pancreatic lipase inhibition by flavonoid **30**.

Uncertainties calculation by the "Jackknife" procedure (95% level of confidance)

Eliminated number	V_{max}	K_m	K_{ic}
1	501,68	23,69	1,65
2	507,97	23,85	1,61
3	484,79	23,64	1,81
4	517,50	28,21	1,86
5	507,26	25,71	1,79
6	507,89	25,81	1,79
7	512,09	26,59	1,78
8	507,59	25,80	1,79
9	504,83	25,43	1,77
10	507,72	25,79	1,79
11	506,94	25,71	1,79
12	513,06	26,94	1,95
13	507,47	25,76	1,79
14	506,37	25,69	1,81
15	507,38	25,75	1,79

V_{max} error:	3,92
K_m error:	0,67
K_{ic} error:	0,04

Figure S8. Error parameters determination (V_{max}, K_m and K_{ic}) for competitive inhibition model of porcine pancreatic lipase by flavonoid **30**, through "Jackknife" procedure.

Flavonoid 38 (Myricetin)

Nonlinear regression using Solver

values of x	values of y	concentration of inhibitor (µM)	slopes (replicates)			standard deviation y_{exp}
12,5	251,53	0	180,926	326,904	246,764	73,11
50	454,64	0	432,175	504,503	427,233	43,26
200	603,49	0	622,151	603,620	584,684	18,73
12,5	181,13	3,75	130,710	244,635	168,030	58,08
50	373,14	3,75	316,114	439,650	363,657	62,31
200	533,31	3,75	428,838	662,031	509,063	118,47
12,5	154,18	7,5	102,990	216,709	142,827	57,70
50	339,11	7,5	304,981	394,065	318,288	48,05
200	496,60	7,5	424,089	617,458	448,254	105,36
12,5	117,37	15	65,230	169,629	117,244	52,20
50	290,37	15	255,443	331,874	283,800	38,64
200	420,48	15	321,547	513,887	426,018	96,29
12,5	90,98	30	43,179	146,832	82,938	52,29
50	231,40	30	191,002	278,199	224,999	43,95
200	351,00	30	255,690	462,238	335,063	104,19

Figure S9. Mean values of the slopes (y values) and respective standard deviations as results of the in vitro inhibition of porcine pancreatic lipase (0.04 mg/mL) by flavonoid **38** (0–30 µM) using three substrate concentrations (x values: 12.5, 50 and 200 µM).

	Without Inhibition	Competitive Inhibition	Noncompetitive Inhibition	Uncompetitive Inhibition	Mixed Inhibition
Equation	$v_{inic} = \frac{V_{max} \times \lvert S \rvert}{K_m + \lvert S \rvert}$	$v_{inic} = \frac{V_{max} \times \lvert S \rvert}{K_m \times (1 + \frac{\lvert I \rvert}{K_{ic}}) + \lvert S \rvert}$	$v_{inic} = \frac{V_{max} \times \lvert S \rvert}{K_m \times (1 + \frac{\lvert I \rvert}{K_{ic}}) + \lvert S \rvert \times (1 + \frac{\lvert I \rvert}{K_{iu}})}$	$v_{inic} = \frac{V_{max}(S)}{K_m + (S) \times (1 + \frac{\lvert I \rvert}{K_{iu}})}$	$v_{inic} = \frac{V_{max} \times \lvert S \rvert}{K_m \times (1 + \frac{\lvert I \rvert}{K_{ic}}) + \lvert S \rvert \times (1 + \frac{\lvert I \rvert}{K_{iu}})}$

$(y_{exp} - y_{cal})^2$	Ycalc	$(y_{exp} - y_{cal})^2$	Ycalc	$(y_{exp} - y_{cal})^2$	Ycalc	$(y_{exp} - y_{cal})^2$	Ycalc	$(y_{exp} - y_{cal})^2$	Ycalc
37552,8287	156,9021	10862,2041	259,1332	17603,5475	203,5218	25373,5455	181,5676	11760,7069	232,6291
43163,1420	340,0057	3755,8774	452,4970	4964,2512	434,4536	8464,8340	414,9604	3793,1525	450,5133
46400,7462	480,0632	7390,2353	556,2681	729,1645	606,4988	892,5687	611,4569	1397,6946	588,2559
8506,9645	156,9021	6846,5746	186,8947	6756,8389	182,9626	6850,4777	175,2438	6976,6977	189,8808
11059,2432	340,0057	8355,8539	387,1683	8676,4963	390,5663	8077,9276	383,3453	9105,7002	394,2764
36577,4027	480,0632	28131,3589	528,8435	28497,8391	545,2320	28495,6567	545,2015	28184,4578	539,4472
6681,5248	156,9021	6852,3482	146,1520	7091,2609	166,1760	7349,6292	169,3457	6775,6288	160,4046
4620,7960	340,0057	4620,2595	338,3233	5350,4384	354,7323	5495,1286	356,2065	5008,9608	350,5213
23022,3509	480,0632	22365,9914	503,9960	22207,7295	495,2076	22268,1697	491,9008	22208,8144	498,1175
10138,5305	156,9021	6178,8120	101,7774	7042,5886	140,4109	10566,1013	158,6654	5525,6703	122,4023
10376,0561	340,0057	4211,5788	270,1574	3248,4575	299,7321	4392,3951	312,0268	3022,7829	286,8538
29192,3146	480,0632	23396,2133	460,7040	18555,9678	418,4271	18788,0540	411,4511	18936,4873	431,9326
18505,0613	156,9021	7764,0095	63,3243	6255,6907	107,1762	12942,2386	140,8935	5657,7917	83,0505
39248,6761	340,0057	8388,3486	192,5620	3883,6353	228,7867	4902,1547	250,0102	5184,2147	210,4154
71686,1196	480,0632	27045,2562	393,1609	24709,4376	319,3871	26744,6850	310,0385	21996,9467	341,2490
396731,7573	sum	176164,9218	sum	165573,3440	sum	191603,5665	sum	155535,7074	sum

	Without Inhibition	Competitive Inhibition	Noncompetitive Inhibition	Uncompetitive Inhibition	Mixed Inhibition
V_{max} =	556,4717	602,3107	698,7323	726,0609	655,0115
K_m =	31,8327	16,5541	30,4151	37,4856	22,6961
K_{ic} =		5,5279			11,9727
K_{iu} = K_{ic} =			33,3724		
K_{iu} =				25,9872	57,5128

Figure S10. Sum of the squares (sum) of the different models (without inhibition, competitive inhibition, noncompetitive inhibition, uncompetitive inhibition, and mixed inhibition) determined from the results obtained from porcine pancreatic lipase inhibition by flavonoid **38**.

Models comparison based on F test

	$(y_{exp} - y_{cal})^2$	p	N	N-p	$f_{0,05}$	Fcalc	ΔSS	Δdf	ΔSS / Δdf
Without Inhibition	396731,7573	2	45	43					
Competitive Inhibition	176164,9218	3	45	42	1,667	52,59	0,133	0,024	5,438
Noncompetitive Inhibition	165573,3440	3	45	42	1,671	58,64	0,065	0,024	2,646
Uncompetitive Inhibition	191603,5665	3	45	42	1,671	44,96	0,232	0,024	9,508
Mixed Inhibition	155535,7074	4	45	41	1,678	31,79			
	Criterium 1					Criterium 2			Criterium 3
	Lower ΔSS for the same N-p					Higher Fcalc			Evaluate if mixed inhibition is better than other inhibition

If Δ SS/Δ df value is higher than $f_{0,05}$ value, mixed inhibition is prefered

Comparison based on Akaike:

	AIC c	ΔAICc			
Without Inhibition	415,3540				
Competitive Inhibition	381,2155	-34,14			
Noncompetitive Inhibition	378,4252	-36,93			
Uncompetitive Inhibition	384,9958	-30,36			
Mixed Inhibition	378,1220	-37,23	-3,09	-0,30	-6,87

Figure S11. Comparison of the different models (without inhibition, competitive inhibition, noncompetitive inhibition, uncompetitive inhibition, and mixed inhibition), based on the porcine pancreatic lipase inhibition by flavonoid **38**.

Uncertainties calculation by the "Jackknife" procedure (95% level of confidance)

Eliminated number	V_{max}	K_m	K_{ic}	K_{iu}
1	673,24	26,90	16,52	49,46
2	654,92	23,02	12,36	57,39
3	628,81	20,61	10,83	71,96
4	652,72	22,20	11,77	57,76
5	655,75	22,13	11,74	56,79
6	659,20	23,03	12,10	56,57
7	654,42	22,58	12,13	56,89
8	655,40	22,61	12,24	56,80
9	655,53	22,74	11,97	57,64
10	655,26	22,77	12,31	56,47
11	654,87	22,64	11,74	57,90
12	654,50	22,64	11,64	62,80
13	654,10	22,47	11,41	59,36
14	654,19	22,03	10,20	60,62
15	661,71	23,25	12,83	48,51

V_{max} error:	4,89
K_m error:	0,72
K_{ic} error:	0,76
K_{iu} error:	2,95

Figure S12. Error parameters determination (V_{max}, K_m, K_{ic} and K_{iu}) for mixed inhibition model of porcine pancreatic lipase by flavonoid **38**, through "Jackknife" procedure.

Flavonoid 41 (Epigallocatechin gallate, EGCG)

Nonlinear regression using Solver

values of x	values of y	concentration of inhibitor (µM)	slopes (replicates)			standard deviation y_{exp}
12,5	331,00	0	341,407	349,187	302,410	25,06
50	496,80	0	463,904	546,262	480,228	43,61
200	654,50	0	664,102	684,124	615,285	35,41
12,5	179,64	7,5	173,842	217,284	147,793	35,11
50	390,58	7,5	372,987	453,208	345,533	55,95
200	559,36	7,5	510,884	660,694	506,514	87,78
12,5	147,47	15	149,785	173,166	119,451	26,93
50	361,97	15	346,551	417,383	321,986	49,53
200	551,27	15	546,781	636,610	470,405	83,19
12,5	120,12	30	134,761	121,959	103,651	15,64
50	366,38	30	407,049	401,272	290,810	65,51
200	567,42	30	579,108	661,391	461,773	100,32
12,5	92,55	60	105,816	89,285	82,558	11,97
50	277,96	60	271,122	277,987	284,759	6,82
200	545,91	60	535,869	625,866	475,996	75,44

Figure S13. Mean values of the slopes (y values) and respective standard deviations as results of the in vitro inhibition of porcine pancreatic lipase (0.04 mg/mL) by flavonoid **41** (EGCG) (0 – 60 µM) using three substrate concentrations (x values: 12.5, 50 and 200 µM).

Without Inhibition	Competitive Inhibition	Noncompetitive Inhibition	Uncompetitive Inhibition	Mixed Inhibition
$v_{inic} = \frac{V_{max} \times \lvert S\rvert}{K_m + \lvert S\rvert}$	$v_{inic} = \frac{V_{max} \times \lvert S\rvert}{K_m \times (1 + \frac{\lvert I\rvert}{K_{ic}}) + \lvert S\rvert}$	$v_{inic} = \frac{V_{max} \times \lvert S\rvert}{K_m \times (1 + \frac{\lvert I\rvert}{K_{ic}}) + \lvert S\rvert \times (1 + \frac{\lvert I\rvert}{K_{iu}})}$	$v_{inic} = \frac{V_{max}(S)}{K_m + (S) \times (1 + \frac{\lvert I\rvert}{K_{iu}})}$	$v_{inic} = \frac{V_{max} \times \lvert S\rvert}{K_m \times (1 + \frac{\lvert I\rvert}{K_{ic}}) + \lvert S\rvert \times (1 + \frac{\lvert I\rvert}{K_{iu}})}$

$(y_{exp} - y_{cal})^2$	Ycalc	$(y_{exp} - y_{cal})^2$	Ycalc	$(y_{exp} - y_{cal})^2$	Ycalc	$(y_{exp} - y_{cal})^2$	Ycalc	$(y_{exp} - y_{cal})^2$	Ycalc
82035,2070	166,9091	16435,9173	259,8689	58583,1013	192,7665	71856,4506	177,5956	16435,6459	259,8696
40946,1863	385,5282	4186,7755	485,4915	14524,3421	437,0176	22814,2477	417,1928	4186,7231	485,4923
22321,2189	573,2353	6061,8939	620,0832	3170,7808	639,6355	4380,8699	629,5152	6061,7561	620,0839
2951,1181	166,9091	5719,3232	212,5760	2530,5805	184,3180	2512,1221	175,6730	5719,3805	212,5763
6337,4537	385,5282	13528,0813	439,7934	8494,9778	417,8644	7044,4624	406,7361	13528,1997	439,7938
15988,4440	573,2353	20406,6975	600,1704	23597,6527	611,6021	21937,8082	606,0066	20406,7846	600,1708
2584,6592	166,9091	4595,8811	179,8462	3993,1880	176,5791	3529,6137	173,7916	4595,9026	179,8463
6571,5587	385,5282	9703,4179	401,9581	9318,3476	400,3195	8543,8487	396,7909	9703,4518	401,9583
15290,2584	573,2353	16584,0337	581,4968	17445,6175	585,9227	17094,4516	584,1907	16584,0482	581,4968
7055,6096	166,9091	1395,1896	137,5039	5978,3403	162,8997	7996,0398	170,1472	1395,1846	137,5039
9682,5471	385,5282	10228,6767	342,9503	8608,0031	369,3072	9008,1001	378,2914	10228,7019	342,9501
20229,7618	573,2353	21327,5738	547,4313	22297,9805	540,5320	21643,0840	544,9545	21327,6192	547,4309
16872,9926	166,9091	289,0909	93,4846	7341,3036	141,0464	15301,2705	163,2985	289,0901	93,4845
34808,3067	385,5282	587,8372	265,1127	5336,6741	319,7638	13993,5402	346,0260	587,8714	265,1122
13621,7189	573,2353	20753,4931	490,0183	29583,1875	468,0184	24248,2670	480,4211	20753,8359	490,0173
297297,0408	sum	151803,8827	sum	220804,0775	sum	251904,1763	sum	151804,1957	sum

Without Inhibition		Competitive Inhibition		Noncompetitive Inhibition		Uncompetitive Inhibition		Mixed Inhibition	
V_{max} =	684,2921	V_{max} =	683,2190	V_{max} =	756,5586	V_{max} =	758,1266	V_{max} =	683,2195
K_m =	38,7474	K_m =	20,3636	K_m =	36,5593	K_m =	40,8605	K_m =	20,3636
		K_{ic} =	20,8891	K_{iu}= K_{ic}=	163,6265	K_{iu} =	160,5378	K_{ic} =	20,8889
								K_{iu} =	18317408

Figure S14. Sum of the squares (sum) of the different models (without inhibition, competitive inhibition, noncompetitive inhibition, uncompetitive inhibition, and mixed inhibition) determined from the results obtained from porcine pancreatic lipase inhibition by flavonoid **41** (EGCG).

Models comparison based on F test

	$(y_{exp} - y_{cal})^2$	p	N	N-p	$f_{0,05}$	Fcalc	ΔSS	Δdf	ΔSS / Δdf
Without Inhibition	297297,0408	2	45	43					
Competitive Inhibition	151803,8827	3	45	42	1,667	40,25	0,000	0,024	0,000
Noncompetitive Inhibition	220804,0775	3	45	42	1,671	14,55	0,455	0,024	18,636
Uncompetitive Inhibition	251904,1763	3	45	42	1,671	7,57	0,659	0,024	27,035
Mixed Inhibition	151804,1957	4	45	41	1,678	19,65			
	Criterium 1					Criterium 2			Criterium 3
	Lower ΔSS for the same N-p					Higher Fcalc			Evaluate if mixed inhibition is better than other inhibition

If Δ SS/Δ df value is higher than $f_{0,05}$ value, mixed inhibition is prefered

Comparison based on Akaike:

	AIC c	ΔAICc			
Without Inhibition	402,3702				
Competitive Inhibition	374,5181	-27,85			
Noncompetitive Inhibition	391,3790	-10,99			
Uncompetitive Inhibition	397,3088	-5,06			
Mixed Inhibition	377,0292	-25,34	2,51	-14,35	-20,28

Figure S15. Comparison of the different models (without inhibition, competitive inhibition, noncompetitive inhibition, uncompetitive inhibition, and mixed inhibition), based on the porcine pancreatic lipase inhibition by flavonoid **41** (EGCG).

Uncertainties calculation by the "Jackknife" procedure (95% level of confidance)

Eliminated number	V_{max}	K_m	K_{ic}
1	710,45	29,44	36,28
2	682,90	21,15	22,46
3	664,44	19,46	21,77
4	675,71	18,66	19,24
5	683,21	18,91	19,00
6	702,71	21,58	20,86
7	677,64	19,46	20,64
8	682,05	19,69	20,85
9	695,51	21,31	21,47
10	681,05	20,21	21,36
11	684,89	20,38	19,70
12	676,81	19,67	19,91
13	683,15	20,37	20,93
14	684,61	20,13	19,54
15	671,21	18,01	15,96

V_{max} error:	6,73
K_m error:	1,53
K_{ic} error:	2,55

Figure S16. Error parameters determination (V_{max}, K_m and K_{ic}) for competitive inhibitions model of porcine pancreatic lipase by flavonoid **41** (EGCG), through "Jackknife" procedure.

HUMAN PANCREATIC LIPASE
Flavonoid 30

Nonlinear regression using Solver

values of x	values of y	concentration of inhibitor (µM)	slopes (replicates)			standard deviation y_{exp}
12,5	133,69	0	131,200	138,144	131,740	3,86
50	189,02	0	191,000	205,453	170,614	17,50
200	261,16	0	266,744	266,782	249,952	9,71
12,5	64,44	1,825	58,003	75,972	59,343	10,01
50	140,93	1,825	121,715	144,385	156,704	17,75
200	190,69	1,825	166,991	216,070	189,014	24,58
12,5	42,88	3,75	40,893	43,116	44,634	1,88
50	108,79	3,75	89,372	121,378	115,628	17,06
200	156,90	3,75	152,232	169,191	149,268	10,75
12,5	25,77	10	22,088	29,532	25,701	3,72
50	74,00	10	62,640	91,924	67,445	15,70
200	133,80	10	132,707	141,239	127,459	6,95

Figure S17. Mean values of the slopes (y values) and respective standard deviations as results of the in vitro inhibition of human pancreatic lipase (2 U/mL) by flavonoid **30** (0–10 µM) using three substrate concentrations (x values: 12.5, 50 and 200 µM).

	Without Inhibition		Competitive Inhibition		Noncompetitive Inhibition		Uncompetitive Inhibition		Mixed Inhibition	
Equation	$v_{inic} = \frac{V_{max} \times [S]}{K_m + [S]}$		$v_{inic} = \frac{V_{max} \times [S]}{K_m \times (1 + \frac{[I]}{K_{ic}}) + [S]}$		$v_{inic} = \frac{V_{max} \times [S]}{K_m \times (1 + \frac{[I]}{K_{ic}}) + [S] \times (1 + \frac{[I]}{K_{iu}})}$		$v_{inic} = \frac{V_{max}(S)}{K_m + (S) \times (1 + \frac{[I]}{K_{iu}})}$		$v_{inic} = \frac{V_{max} \times [S]}{K_m \times (1 + \frac{[I]}{K_{ic}}) + [S] \times (1 + \frac{[I]}{K_{iu}})}$	
	$(y_{exp} - y_{cal})^2$	Ycalc	$(y_{exp} - y_{cal})^2$	Ycalc	$(y_{exp} - y_{cal})^2$	Ycalc	$(y_{exp} - y_{cal})^2$	Ycalc	$(y_{exp} - y_{cal})^2$	Ycalc
	17836,5771	56,6520	42,7953	131,6166	4115,3241	96,7917	7133,0894	85,0351	303,5392	124,1431
	11570,4743	128,5858	980,5831	200,0954	677,1354	193,6552	685,6770	184,0917	1187,5845	202,8648
	16075,9201	188,3868	3098,5297	230,0139	213,4381	258,2708	194,5244	259,7314	1397,4830	241,0838
	382,3310	56,6520	244,5524	60,6031	508,3051	74,5702	664,0356	76,8709	236,0846	67,8882
	1087,4570	128,5858	648,6476	138,4396	834,7070	149,1957	859,2637	149,6771	671,7425	144,6661
	1224,5328	188,3868	1733,1751	203,9151	1414,5266	198,9768	1296,7631	196,1128	1571,6012	201,6917
	575,9983	56,6520	61,4849	38,6225	889,6194	60,0327	2181,3079	69,8020	35,0282	45,9332
	1757,5771	128,5858	638,0329	104,4815	966,5110	120,1099	1372,6522	125,0241	597,6854	111,0591
	3205,9340	188,3868	2139,4904	182,1186	263,5603	160,1861	234,4087	155,8478	919,1010	172,0407
	2888,1213	56,6520	221,5092	17,7364	390,0204	36,7631	2376,2814	53,7532	61,7283	22,4065
	9431,1282	128,5858	1246,1443	58,1615	493,8934	73,5536	660,1668	81,4613	836,3811	63,3089
	9035,3609	188,3868	102,5947	135,1985	3921,4997	98,0957	4966,5305	93,5119	999,4288	116,4553
	75071,4122	sum	11157,5395	sum	14688,5404	sum	22624,7006	sum	8817,3880	sum
	V_{max} =	222,9489	V_{max} =	242,0792	V_{max} =	290,5906	V_{max} =	300,9494	V_{max} =	257,2381
	K_m =	36,6927	K_m =	10,4909	K_m =	25,0278	K_m =	31,7390	K_m =	13,4014
			K_{ic} =	0,7107	K_{iu} = K_{ic} =	6,1243	K_{iu} =	4,8553	K_{ic} =	1,2159
									K_{iu} =	16,9264

Figure S18. Sum of the squares (sum) of the different models (without inhibition, competitive inhibition, noncompetitive inhibition, uncompetitive inhibition, and mixed inhibition) determined from the results obtained from human pancreatic lipase inhibition by flavonoid **30**.

Models comparison based on F test

	$(y_{exp} - y_{cal})^2$	p	N	N-p	$f_{0,05}$	Fcalc	ΔSS	Δdf	ΔSS / Δdf
Without Inhibition	75071,4122	2	36	34					
Competitive Inhibition	11157,5395	3	36	33	1,783	189,03	0,265	0,031	8,493
Noncompetitive Inhibition	14688,5404	3	36	33	1,788	135,66	0,666	0,031	21,308
Uncompetitive Inhibition	22624,7006	3	36	33	1,788	76,50	1,566	0,031	50,109
Mixed Inhibition	8817,3880	4	36	32	1,799	120,22			
	Criterium 1					Criterium 2			Criterium 3
	Lower ΔSS for the same N-p					Higher Fcalc			Evaluate if mixed inhibition is better than other inhibition

If Δ SS/Δ df value is higher than $f_{0,05}$ value, mixed inhibition is prefered

Comparison based on Akaike:

	AIC c	ΔAICc			
Without Inhibition	281,8422				
Competitive Inhibition	215,7208	-66,12			
Noncompetitive Inhibition	225,6191	-56,22			
Uncompetitive Inhibition	241,1702	-40,67			
Mixed Inhibition	209,9096	-71,93	-5,81	-15,71	-31,26

Figure S19. Comparison of the different models (without inhibition, competitive inhibition, noncompetitive inhibition, uncompetitive inhibition, and mixed inhibition), based on the human pancreatic lipase inhibition by flavonoid **30**.

Uncertainties calculation by the "Jackknife" procedure (95% level of confidance)

Eliminated number	V_{max}	K_m	K_{ic}	K_{iu}
1	279,76	24,52	2,77	11,08
2	263,30	12,55	1,06	15,25
3	214,33	7,59	0,77	17,88
4	256,90	13,27	1,24	16,37
5	257,59	13,38	1,24	16,52
6	262,61	14,20	1,24	17,92
7	257,26	13,43	1,25	16,36
8	257,27	13,43	1,25	16,67
9	258,18	13,53	1,15	25,57
10	257,13	13,34	1,19	17,33
11	257,66	13,21	1,09	17,90
12	268,19	15,10	1,55	8,47

V_{max} error:	9,64
K_m error:	2,41
K_{ic} error:	0,31
K_{iuc} error:	2,60

Figure S20. S. Error parameters determination (V_{max}, K_m and K_{ic}) for mixed inhibition model of human pancreatic lipase by flavonoid **30**, through "Jackknife" procedure.

Flavonoid 38 (Myricetin)

Nonlinear regression using Solver

values of x	values of y	concentration of inhibitor (µM)	slopes (replicates)				standard deviation y_{exp}
12,5	134,06	0	148,717	122,449	142,786	122,280	13,72
50	186,52	0	195,327	176,848	187,389		9,27
200	230,27	0	216,221	247,667	226,928		15,99
12,5	118,46	6,25	139,618	111,795	124,546	97,882	17,82
50	171,93	6,25	205,579	159,596	150,618		29,48
200	197,36	6,25	223,582	180,875	220,523	164,446	29,32
12,5	112,07	12,5	116,329	110,329	109,555		3,71
50	166,25	12,5	139,271	181,783	177,697		23,45
200	184,56	12,5	232,497	154,054	167,139		42,02
12,5	101,80	25	112,215	111,984	105,278	77,739	16,36
50	146,98	25	174,881	159,784	151,610	101,641	31,73
200	172,37	25	182,973	155,952	196,006	154,543	20,48
12,5	75,96	50	88,951	72,045	66,893		11,54
50	108,15	50	117,298	112,363	94,785		11,83
200	130,19	50	121,344	137,785	131,430		8,29

Figure S21. Mean values of the slopes (y values) and respective standard deviations as results of the in vitro inhibition of human pancreatic lipase (2 U/mL) by flavonoid **38** (0–50 µM) using three substrate concentrations (x values: 12.5, 50 and 200 µM).

Without Inhibition	Competitive Inhibition	Noncompetitive Inhibition	Uncompetitive Inhibition	Mixed Inhibition
$v_{inic} = \frac{V_{max} \times \vert S \vert}{K_m + \vert S \vert}$	$v_{inic} = \frac{V_{max} \times \vert S \vert}{K_m \times (1 + \frac{\vert I \vert}{K_{ic}}) + \vert S \vert}$	$v_{inic} = \frac{V_{max} \times \vert S \vert}{K_m \times (1 + \frac{\vert I \vert}{K_{ic}}) + \vert S \vert \times (1 + \frac{\vert I \vert}{K_{iu}})}$	$v_{inic} = \frac{V_{max}(S)}{K_m + (S) \times (1 + \frac{\vert I \vert}{K_{iu}})}$	$v_{inic} = \frac{V_{max} \times \vert S \vert}{K_m \times (1 + \frac{\vert I \vert}{K_{ic}}) + \vert S \vert \times (1 + \frac{\vert I \vert}{K_{iu}})}$

$(y_{exp} - y_{cal})^2$	Ycalc	$(y_{exp} - y_{cal})^2$	Ycalc	$(y_{exp} - y_{cal})^2$	Ycalc	$(y_{exp} - y_{cal})^2$	Ycalc	$(y_{exp} - y_{cal})^2$	Ycalc
3177,8709	108,4977	883,7650	142,9912	613,9330	130,5445	913,1081	124,7232	609,4191	130,7089
2359,9384	159,5147	287,9779	180,3001	314,9548	193,4276	288,4245	192,7545	314,9241	193,4268
7864,2894	180,7641	4705,3239	192,8816	833,3013	219,9102	661,6750	223,1897	840,6569	219,7925
1349,5866	108,4977	993,1697	121,6459	966,6529	120,3362	952,6029	118,3793	967,5063	120,3922
2201,0765	159,5147	1742,0914	170,8498	1860,3492	178,3019	1849,5054	178,0116	1860,2605	178,2996
3680,4327	180,7641	2791,5867	190,0698	2693,9986	202,7136	2738,1103	203,6594	2692,1432	202,6701
65,8009	108,4977	143,7644	105,8455	28,1369	111,6086	28,4996	112,6496	28,2043	111,5849
1236,2800	159,5147	1146,0287	162,3408	1102,4980	165,3703	1102,5328	165,3637	1102,5162	165,3668
3575,3653	180,7641	3555,1738	187,3389	3567,7318	188,0115	3554,0748	187,2721	3567,9646	188,0227
982,4365	108,4977	2068,3916	84,0193	878,3416	97,4702	806,4771	102,7072	882,8283	97,3427
3648,0466	159,5147	3021,1920	147,6351	3045,6346	144,4214	3038,6570	144,7889	3045,7341	144,4166
1540,3825	180,7641	1637,6943	182,1058	1525,6948	164,1945	1747,3983	161,3123	1520,3083	164,2773
3441,8232	108,4977	1080,7542	59,4862	276,0706	77,7673	651,7165	87,2975	273,8318	77,5471
8195,4710	159,5147	1131,0092	124,9906	430,3979	115,2277	462,0967	115,9384	430,1465	115,2218
7811,8109	180,7641	5501,2659	172,4703	139,4781	131,0038	182,8451	126,2974	140,2656	131,1511
51130,6114	sum	30689,1887	sum	18277,1742	sum	18977,7240	sum	18276,7099	sum

Without Inhibition		Competitive Inhibition		Noncompetitive Inhibition		Uncompetitive Inhibition		Mixed Inhibition	
V_{max} =	189,1638	V_{max} =	197,4749	V_{max} =	230,4262	V_{max} =	235,5893	V_{max} =	230,2544
K_m =	9,2935	K_m =	4,7629	K_m =	9,5640	K_m =	11,1112	K_m =	9,5198
		K_{ic} =	9,8272	K_{iu}= K_{ic}=	73,6752	K_{iu} =	61,7438	K_{ic} =	71,4560
								K_{iu} =	74,1030

Figure S22. Sum of the squares (sum) of the different models (without inhibition, competitive inhibition, noncompetitive inhibition, uncompetitive inhibition, and mixed inhibition) determined from the results obtained from human pancreatic lipase inhibition by flavonoid **38**.

Models comparison based on F test

	$(y_{exp} - y_{cal})^2$	p	N	N-p	$f_{0,05}$	Fcalc	ΔSS	Δdf	ΔSS / Δdf
Without Inhibition	51130,6114	2	51	49					
Competitive Inhibition	30689,1887	3	51	48	1,612	31,97	0,679	0,021	31,920
Noncompetitive Inhibition	18277,1742	3	51	48	1,615	86,28	0,000	0,021	0,001
Uncompetitive Inhibition	18977,7240	3	51	48	1,615	81,32	0,038	0,021	1,803
Mixed Inhibition	18276,7099	4	51	47	1,621	42,24			
	Criterium 1					Criterium 2			Criterium 3
	Lower ΔSS for the same N-p					Higher Fcalc			Evaluate if mixed inhibition is better than other inhibition

If Δ SS/Δ df value is higher than $f_{0.05}$ value, mixed inhibition is prefered

Comparison based on Akaike:

	AIC c	ΔAICc			
Without Inhibition	358,9158				
Competitive Inhibition	335,2252	-23,69			
Noncompetitive Inhibition	308,7940	-50,12			
Uncompetitive Inhibition	310,7123	-48,20			
Mixed Inhibition	311,2360	-47,68	-23,99	2,44	0,52

Figure S23. Comparison of the different models (without inhibition, competitive inhibition, noncompetitive inhibition, uncompetitive inhibition, and mixed inhibition), based on the human pancreatic lipase inhibition by flavonoid **38**.

Uncertainties calculation by the "Jackknife" procedure (95% level of confidance)

Eliminated number	V_{max}	K_m	$K_{iu} = K_{ic}$
1	230,48	9,88	75,08
2	232,51	9,56	71,05
3	224,50	9,10	79,53
4	230,24	9,43	73,45
5	231,47	9,56	72,86
6	233,09	9,83	72,36
7	230,48	9,58	73,66
8	230,35	9,57	73,65
9	231,21	9,67	73,76
10	231,38	9,81	72,61
11	230,47	9,57	72,84
12	229,38	9,27	70,43
13	230,07	9,51	74,24
14	229,55	9,55	77,18
15	230,38	9,58	74,16

V_{max} error:	1,06
K_m error:	0,11
$K_{iu} = K_{ic}$ error:	1,24

Figure S24. Error parameters determination (V_{max}, K_m, K_{ic} and K_{iu}) for noncompetitive inhibition model of human pancreatic lipase by flavonoid **38**, through "Jackknife" procedure.

Molecular docking

Table S1. Molecular docking scores for the interaction of all tested flavonoids with human pancreatic lipase obtained with GNINA (CNN Affinity), from lowest to highest.

COMPOUND	GNINA Score (CNN affinity)	COMPOUND	GNINA Score (CNN affinity)	COMPOUND	GNINA Score (CNN affinity)
3	5.034	40	5.739	17	6.099
1	5.069	22	5.792	12	6.116
26	5.150	20	5.793	42	6.131
25	5.268	3	5.823	48	6.138
5	5.401	45	5.823	24	6.142
13	5.462	19	5.872	**38***	**6.167***
**15***	**5.493***	37	5.872	27	6.199
6	5.547	21	5.891	34	6.213
16	5.572	10	5.892	23	6.221
8	5.584	7	5.893	32	6.313
39	5.638	47	5.893	**41***	**6.328***
**36***	**5.639***	44	5.951	33	6.341
11	5.645	4	5.954	35	6.655
2	5.656	46	5.982	**28***	**6.796***
9	5.692	18	5.989	**29***	**7.015***
31	5.696	43	6.027	**30***	**7.455***
**14***	**5.728***				

* Flavonoids that demonstrated catalytic inhibition against human pancreatic lipase.
